# Clinical features, myocardial strain and tissue characteristics of heart failure with preserved ejection fraction in patients with obesity: A prospective cohort study

**DOI:** 10.1016/j.eclinm.2022.101723

**Published:** 2022-11-03

**Authors:** Jian He, Wenjing Yang, Weichun Wu, Xiaoxin Sun, Shuang Li, Gang Yin, Baiyan Zhuang, Jing Xu, Di Zhou, Yuhui Zhang, Yining Wang, Leyi Zhu, Piyush Sharma, Arlene Sirajuddin, Zhongzhao Teng, Faraz Kureshi, Shihua Zhao, Minjie Lu

**Affiliations:** a Department of Magnetic Resonance Imaging, Fuwai Hospital, National Centre for Cardiovascular Diseases, Chinese Academy of Medical Sciences and Peking Union Medical College, Beijing, China; b Department of Echocardiography, Fuwai Hospital, National Centre for Cardiovascular Diseases, Chinese Academy of Medical Sciences and Peking Union Medical College, Beijing, China; c Department of Nuclear Medicine, Fuwai Hospital, National Centre for Cardiovascular Diseases, Chinese Academy of Medical Sciences and Peking Union Medical College, Beijing, China; d Department of Heart Failure Centre, Fuwai Hospital, National Centre for Cardiovascular Diseases, Chinese Academy of Medical Sciences and Peking Union Medical College, Beijing, China; e Saint James School of Medicine, Park Ridge, IL, 60068, USA; f Department of Health and Human Services, National Heart, Lung, and Blood Institute, National Institutes of Health, Bethesda, Md, USA; g Department of Radiology, University of Cambridge, Cambridge, UK; h Axis Cardiovascular and Axis Cardiovascular Advanced Imaging, St David's Healthcare, Austin, Tex, USA; i Key Laboratory of Cardiovascular Imaging (Cultivation), Chinese Academy of Medical Sciences, Beijing, China

**Keywords:** Heart failure with preserved ejection fraction, Obesity, Cardiovascular magnetic resonance, Myocardial strain, Myocardial fibrosis

## Abstract

**Background:**

The pathophysiology and subsequent myocardial dysfunction of heart failure with preserved ejection fraction (HFpEF) with comorbid obesity has not been extensively described. This study aimed to investigate the clinical features and cardiovascular magnetic resonance (CMR) derived myocardial strain and tissue characteristics in patients with HFpEF and comorbid obesity phenotype.

**Methods:**

In this prospective cohort study, we included consecutive patients admitted to Fuwai hospital in China who underwent CMR. Patients with HFpEF or obesity were diagnosed with demographic data, clinical presentation, laboratory test, and echocardiography or CMR imaging. The key exclusion criteria were cardiomyopathy, primary valvular heart disease, and significant coronary artery disease. Participant data were obtained from the electronic medical records database or inquiry. Comparisons of clinical features and CMR derived structural and functional parameters amongst different groups were made using one-way analysis of variance, or χ^2^ tests, and post hoc Bonferroni analysis where appropriate.

**Findings:**

Between January 1, 2019 and July 31, 2021, 280 participants (108 patients with HFpEF and obesity, 50 patients with HFpEF and normal weight, 72 patients with obesity, and 50 healthy controls) were enrolled. Compared with patients with HFpEF and normal weight, patients with HFpEF and obesity were younger males, and had higher plasma volume, uric acid and hemoglobin levels, yet less often atrial fibrillation, and lower NT-proBNP levels, and had higher left ventricular mass index, end-diastole/systole volume index, lower left atrial volume index, and worse myocardial strains (all p ≤ 0.05), but no remarkable difference in late gadolinium enhancement (LGE) presence and extracellular volume fraction (ECV). After adjusting for age, atrial fibrillation, and coronary artery disease, only global longitudinal strain (GLS, p = 0.031) and early-diastolic global longitudinal strain rate (eGLSR, p = 0.043) were considerably worse in patients with HFpEF and obesity versus patients with HFpEF and normal weight. Furthermore, early-diastolic strain rates showed no linear association with ECV in patients with HFpEF and obesity. Moreover, GLS demonstrated the highest diagnostic ability when compared with traditional CMR structural parameters and ECV to diagnose patients with HFpEF and obesity in the setting of obesity.

**Interpretation:**

Higher systemic inflammation, and worse GLS and eGLSR may be the distinct features of obesity-related HFpEF phenotype; strains and ECV may represent different mechanisms of HFpEF with obesity, deserving further study.

**Funding:**

The Construction Research Project of Key Laboratory (Cultivation) of Chinese Academy of Medical Sciences (2019PT310025); 10.13039/501100001809National Natural Science Foundation of China (81971588); Capital’s Funds for Health Improvement and Research (CFH 2020-2-4034); Youth Key Program of High-level Hospital Clinical Research (2022-GSP-QZ-5).

**Research in context**

**Evidence before this study**

We searched PubMed for papers published between database inception up to July 31, 2022. We used the search terms (“HFpEF” OR “heart failure with preserved ejection fraction”), and (“obese” OR “obesity”), and found that several previous studies of high quality have delineated the clinical presentation of patients with heart failure with preserved ejection fraction (HFpEF) and obesity. However, scarce studies were focused on the specific cardiovascular magnetic resonance (CMR) derived quantitative cardiac dysfunction and tissue characteristics of patients with HFpEF and obesity in comparison to patients with HFpEF and normal weight or patients with obesity or controls, thus, making the patients with HFpEF and obesity subgroup still a gap without a deep understanding of the pathophysiology in this specific population.

**Added value of this study**

Based on the data from our study, we systemically reported the detailed clinical and laboratory phenotyping along with CMR functional and tissue characteristics for the poorly described population of patients with HFpEF and obesity, innovatively compared with patients with HFpEF and normal weight, patients with obesity, and control individuals. Compared with patients with HFpEF and normal weight, patients with HFpEF and obesity phenotype were younger, higher plasma volume, more systemic inflammation, less atrial fibrillation, more impaired cardiac function, but similar fibrosis. Furthermore, global longitudinal strain and early-diastolic longitudinal strain rate outperformed structural parameters in illustrating the abnormalities of patients with HFpEF and obesity independent of age, atrial fibrillation, and coronary artery disease. Strains and ECV may represent different mechanisms of patients with HFpEF and obesity.

**Implications of all the available evidence**

Our findings indicated the application of strain parameters for assessing clinical presentation and may become an accessible and promising parameter in monitoring and early diagnosis of patients with HFpEF and obesity. We have now merely embarked on—a potentially revolutionary journey of the application of CMR-FT in the patients with obesity and HFpEF phenotype, and large multicentre studies are needed to establish and verify the role of CMR-feature tracking in the routine clinical decision-making processes.

## Introduction

Obesity is a major epidemic disease worldwide, and it is a common finding in the progression of heart failure with preserved ejection fraction (HFpEF).^1^^,^^2^ Notably, the greatest number of individuals with overweight and obesity worldwide are located in China — approximately half of adults and one-fifth children fall in this category,^3^ placing a high burden on healthcare facilities for the management and diagnosis of HFpEF. Additionally, currently there are no targeted diagnostic criteria and treatment plans due to the complex etiology and multiple phenotypes of HFpEF.^4^, ^5^, ^6^ Several studies have provided evidence for the distinct obesity-related HFpEF phenotype, and its unique pathophysiology.^1^^,^^7^ While these studies were based on the obesity criteria for the European and American population with a body mass index (BMI) greater than 30 kg/m^2^, studies have also reported that the Chinese population have a higher percentage of body fat when compared with western counterparts of equal BMI.^8^ Based on our extensive review, none of the existing studies utilized obesity as defined by the Chinese obesity criteria; in which a BMI greater than 28 kg/m^2^ indicates obesity. Moreover, studies focusing on the specific quantitative cardiac dysfunction and tissue characteristics of patients with HFpEF and obesity when compared to patients with HFpEF and normal weight or patients with obesity or controls were scarce. This further confirmed that amongst Chinese patients with HFpEF and obesity, there remains a large knowledge gap without an adequate understanding of the complete pathophysiology in this specific population.

Cardiovascular magnetic resonance (CMR) has historically been identified as the gold standard for cardiac morphological and functional measurement,^9^ particularly CMR-feature tracking (CMR-FT) and extracellular volume fraction (ECV). These features allow for the identification of subtle and early dysfunction with strain parameters and tissue characteristics.^10^^,^^11^ Studies have also confirmed the prognostic value of CMR-FT derived strain parameters and ECV in the overall HFpEF spectrum.^12^, ^13^, ^14^ However, further studies of cardiac dysfunction based on CMR-FT and ECV of the patients with HFpEF and obesity phenotype when compared to their general HFpEF counterparts were scarce. Hence, this study aimed to illustrate the clinical features and CMR derived left ventricular (LV) remodeling, dysfunction, and tissue characteristics present in the patients with HFpEF and obesity subgroup, as well as quantify these phenotypically diverse patients in comparison to patients with HFpEF and normal weight, or obesity, and control individuals.

## Methods

### Study design and population

As part of the Multimodality Imaging in the Screening, diagnosis and risk StratifictION of HFpEF (MISSION-HFpEF) project (NCT04603404), patients with HFpEF underwent CMR and echocardiography were consecutively included from January 1, 2019 to July 31, 2021 in Fuwai Hospital. The MISSION-HFpEF project was approved by the institution Ethics Review Board of Fuwai Hospital, Beijing, China (Ref. 2019-1307), and written informed consent was obtained from all study participants. In this prospective cohort study, we included patients with HFpEF based on the HFA-PEFF (Heart Failure Association Pretest assessment, Echocardiographic and Natriuretic Peptide Score, Functional testing in Case of uncertainty, Final Aetiology) diagnostic algorithm published by the up-to-date European Society of Cardiology guidelines,^15^ including: (1) symptoms and/or signs of heart failure or New York Heart Association (NYHA) II-IV class; (2) LV ejection fraction, LVEF≥50%; (3) N-terminal pro brain natriuretic peptide (NT-proBNP) >125 pg/ml or BNP >35 pg/ml in sinus rhythm or NT-proBNP >365 pg/ml or BNP >105 pg/ml in atrial fibrillation; (4) relevant structural heart disease, and LV diastolic dysfunction. Definite HFpEF was confirmed with the HFA-PEFF algorithm ≥5 points. Exclusion criteria included cardiomyopathy; primary valvular heart disease; significant coronary artery disease (i.e. angina Canadian Cardiovascular Society ≥ III, unstable angina or acute myocardial infarction<90 days, percutaneous coronary intervention or coronary artery bypass graft<90 days)^16^; idiopathic pulmonary artery hypertension; restrictive pericardial disease; severe arrhythmia; severe renal dysfunction (glomerular filtration rate, GFR<30 ml/min/1.73 m^2^), and poor image quality (could not identify and track the endocardium and epicardium). Finally, enrolled patients were categorized into patients with HFpEF and obesity group (BMI≥28 kg/m^2^) and patients with HFpEF and normal weight group (18.5 ≤ BMI<24 kg/m^2^).^3^^,^^8^ In addition, patients with obesity (BMI≥28 kg/m^2^) and normal weight controls (18.5 ≤ BMI<24 kg/m^2^ and normal imaging report) without heart failure were also consecutively enrolled. Exclusion criteria remained the same as demonstrated above. Participant data were obtained from the electronic medical records database or inquiry. Plasma volume was estimated by (1-hematocrit) × (a + [b × weight in kg]) where a = 1530 for men and 864 for women, and b = 41 for men and 47.9 for women, respectively.^17^

### Echocardiography protocol and analysis

Echocardiography with conventional and tissue Doppler imaging was performed using a commercial ultrasound system (EPIQ 7, Philips Healthcare). All images were digitally stored for offline analysis on a dedicated workstation (QLAB 13.0 system, Philips Healthcare) blinded to the clinical data. Mitral inflow and mitral annulus parameters were measured at rest from the apical four-chamber view by pulse and tissue Doppler imaging.

### CMR protocol and analysis

All individuals underwent CMR using 3T scanners (MR750, General Electric Healthcare, Waukesha, Wisconsin, USA or Ingenia, Philips Healthcare, Best, the Netherlands) during sinus rhythm with retrospective ECG gating and an 8-channel cardiac coil array. For cardiac morphological and functional analysis, steady-state free-precession, breath-hold cines were obtained in 3 long-axis planes and in sequential short-axis slices from the atrioventricular ring to the LV apex. Typical imaging parameters included: TR/TE: 3.0/1.5 msec; matrix size: 192 × 256; slice thickness: 8 mm; integrated parallel imaging technique acceleration factor: 1.5–1.8; temporal resolution: 38–55 msec per frame depending on RR interval.

A modified Look-Locker inversion recovery (MOLLI) sequence was used for T1 mapping as has been previously described.^18^ The MOLLI sequence was performed in the LV short axis for all patients at the base, mid-chamber, and apex. The typical T1 map imaging parameters were: matrix size: 162 × 256; slice thickness: 10 mm; and TR/TE: 2.5/1.0 msec. Late gadolinium enhancement (LGE) image acquisition started at 10 min after administration of gadolinium-based contrast agent by using a gradient spoiled fast low-angle shot sequence with phase-sensitive inversion-recovery technique. LGE CMR was performed in a series of contiguous 8-mm LV short axis slices that covered the entire left ventricle. The inversion time was individually determined per patient to null the myocardial signal.

All CMR images were transferred to an off-line workstation and processed with commercially processing software packages fully blinded to echocardiography results and clinical data. Linear dimensions of the cardiac chambers (LA maximal volumes, LV cardiac index, end-diastolic diameter, volumes, mass, and LVEF) were measured via standard volumetric techniques by CVI42 (Circle Cardiovascular Imaging Inc., Calgary, Alberta, Canada).^19^ LV volume was indexed for body surface area (BSA), and LV mass was indexed for height^2.7^.^20^ We used methods previously described to calculate the ECV by using QMap (Medis QMap).^18^ Hematocrit level was determined for each individual from a venous blood sample drawn less than 24 h prior to the cardiac CMR examination. LGE was semiautomatically quantified by using the fullwidth half-maximum method with manual correction using QMass (Medis QMass). Any obvious blood pool or pericardial partial volume artifacts were manually corrected.

For strain analysis, the endocardial and epicardial contours were automatically detected with manual correction in end-systole and end-diastole using CVI42 via two-dimensional long axis and short axis stacks. Papillary muscles were excluded from the endocardial contour.^10^ Following this, global longitudinal strain (GLS), systolic GLS rate (sGLSR), and early-diastolic GLS rate (eGLSR) were derived from 3 long-axis cines; global circumferential (GCS), radial (GRS) strain, systolic GCS/GRS rate (sGCSR, sGRSR), and early-diastolic GCS/GRS rate (eGCSR, eGRSR) were derived from the short-axis stack.

### Statistical analysis

Sample size estimation was performed using the PASS (version 15.0.5, NCSS). Our study was powered to test the null hypothesis of no significant difference between obesity and normal weight patients with HFpEF groups. For strain analysis, this study was an exploratory investigation. To our knowledge, there is no previously published literature comparing the CMR strain difference between obesity and normal weight patients with HFpEF, we determined the sample size based on the preliminary results in 60 randomly selected patients with HFpEF and obesity (n = 30) and patients with HFpEF and normal weight (n = 30). Group sample sizes of 100 and 46 achieve over 90.0% power for GLS, GCS, GRS to reject the null hypothesis of equal means, and with a significance level (alpha) of 0.05 using a two-sided two-sample unequal-variance t-test. Hence, 108 patients with HFpEF and obesity and 50 patients with HFpEF and normal weight were finally enrolled in our study, which provided at least greater than 90% power for strain analysis.

Variables were depicted as mean ± standard deviation, median (interquartile range), or percentages as appropriate. The main comparisons amongst the patients with HFpEF and obesity cohort, patients with HFpEF and normal weight, patients with obesity, and the controls group were made using one-way analysis of variance, Kruskal–Wallis tests, or χ^2^ tests, and post hoc Bonferroni analysis where appropriate. Analysis of covariance was used to evaluate differences of CMR parameters between obesity and normal weight patients with HFpEF after adjusting for statistically significant baseline parameters (age, atrial fibrillation, and coronary artery disease). In addition, sensitivity analysis of comparisons in all enrolled four groups without atrial fibrillation or coronary artery disease were also performed. Associations between continuous or qualitative measurements were assessed by way of linear regression or Pearson's/Spearman's correlation coefficient (r/ρ). Receiver-operating characteristic (ROC) curves were used to identify parameters that best identified patients with HFpEF and obesity. Linear regression and Bland–Altman analyses were used to determine inter-technique agreement between echocardiography speckle tracking and CMR-FT. Intra- and inter-observer variability of each CMR strain parameters was expressed in terms of intraclass-correlations (ICC), coefficients of variation (CoV) and Bland–Altman plots in 20 randomly chosen patients. Two-sided p < 0.05 was considered statistically significant. All analyses were performed using IBM SPSS Statistics for Windows, v.26.0 (Armonk, NY), the MedCalc Statistical Software, v.19.6.4 (Ostend, Belgium), and OriginPro 2021, v.9.8.

### Role of the funding source

The study sponsors had no role in study design, data collection, data analysis, data interpretation, or writing of the report. All authors had access to the data and had final responsibility for the decision to submit for publication.

## Results

### Baseline data

**Fig. 1:**
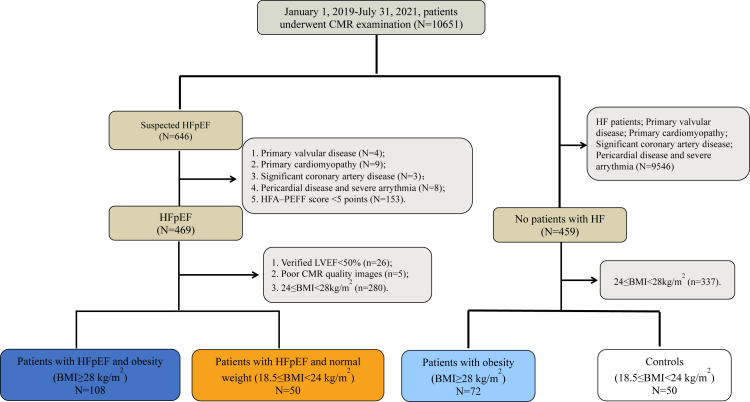
**Patient flowchart.** CMR, cardiovascular magnetic resonance; HFpEF, heart failure with preserved ejection fraction; BMI, body mass index; LVEF, left ventricular ejection fraction.

**Fig. 2:**
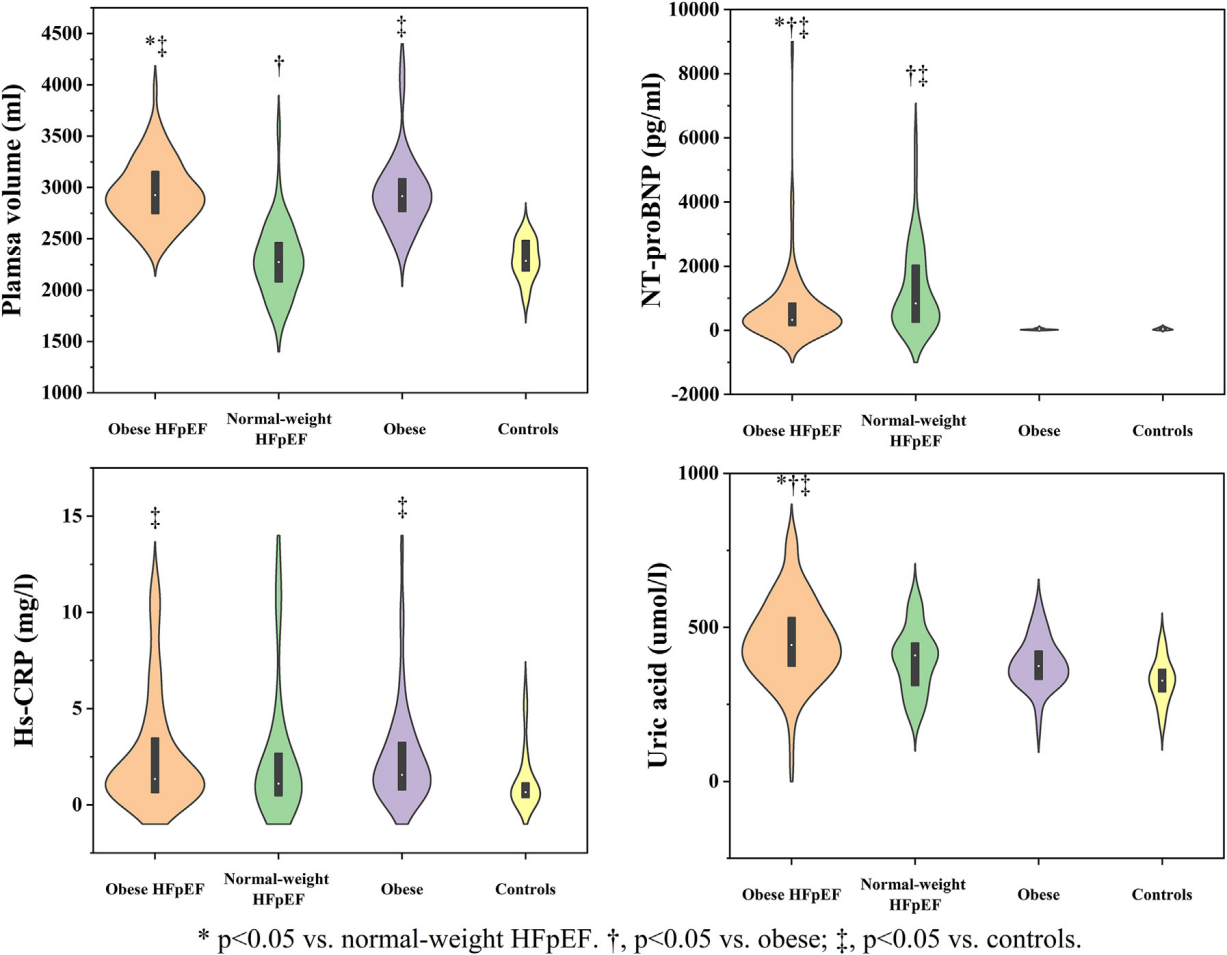
**Violin plots (data distribution plot with median and interquartile range) showing baseline data in four groups.** Obese HFpEF, patients with HFpEF and obesity; normal weight HFpEF, patients with HFpEF and normal weight; obese, patients with obesity; HFpEF, heart failure with preserved ejection fraction; NT-proBNP, N-terminal pro–brain natriuretic peptide; Hs-CRP, hypersensitive-C reactive protein.

A total of 280 individuals were enrolled in this study (patients with HFpEF and obesity n = 108; patients with HFpEF and normal weight n = 50; patients with obesity n = 72; controls n = 50). Reasons for exclusion and baseline data are shown in Fig. 1 and Table 1. No significant differences were observed in NYHA class, hematocrit, blood glucose, and aspirin medication amongst four groups (all p > 0.05). Compared with patients with HFpEF and normal weight, patients with HFpEF and obesity were younger, and had higher plasma volume, uric acid and hemoglobin levels, yet less often atrial fibrillation, and lower NT-proBNP levels. As compared to patients with obesity, patients with HFpEF and obesity showed higher uric acid, creatinine, NT-proBNP levels, yet lower GFR. Compared to controls, patients with obesity were less often female, and had higher diastolic blood pressure, plasma volume, and hypersensitive-C reactive protein (Hs-CRP), and more common comorbidities and drugs usage. Moreover, Hs-CRP level was higher in both patients with HFpEF and obesity and patients with obesity versus controls (Fig. 2).

**Table 1:** Baseline characteristics.

Variables	Patients with HFpEF and obesity (n = 108)	Patients with HFpEF and normal weight (n = 50)	Patients with Obesity (n = 72)	Controls (n = 50)	P-value
Age (years)	46 ± 14∗	64 ± 10^†‡^	45 ± 11	44 ± 10	**<0.0001**
Male (%)	75.0^‡^	56.0	73.6^‡^	44.0	**0.0003**
Female (%)	25.0^‡^	44.0	26.4^‡^	56.0	**0.0003**
Body mass index (kg/m^2^)	31 ± 2∗^‡^	22 ± 2^†^	31 ± 3^‡^	22 ± 1	**<0.0001**
NYHA class III/IV (%)	38.0	32.0	0	0	0**.**21
Systolic BP (mmHg, n = 259)	134 ± 24	132 ± 19	140 ± 17^‡^	124 ± 15	**0.0038**
Diastolic BP (mmHg, n = 259)	85 ± 16^‡^	79 ± 12	85 ± 11^‡^	77 ± 10	**0.0016**
Estimated PV (ml, n = 212)	2954 ± 308∗^‡^	2298 ± 356^†^	2960 ± 364^‡^	2302 ± 191	**<0.0001**
Atrial fibrillation (%)	25.0∗^†‡^	50.0^†‡^	2.8	0	**<0.0001**
Diabetes (%)	28.7^‡^	26.0^‡^	25.0^‡^	4.0	**0.0055**
Hypertension (%)	91.7^†‡^	78.0^‡^	76.4^‡^	14.0	**<0.0001**
Coronary artery disease (%)	13.0^‡^	26.0^†‡^	4.2	0	**<0.0001**
SAHS (%)	13.0^‡^	7.5	23.3^‡^	0	**0.0010**
Dyslipidemia (%)	45.4	44.0	51.4^‡^	26.0	**0.042**
Uric acid (μmol/l, n = 220)	455.2 ± 133.9∗^†‡^	394.4 ± 103.5	382.4 ± 82.5	326.9 ± 70.6	**<0.0001**
HsCRP (mg/l, n = 214)	1.35 (0.64,3.48)^‡^	1.11 (0.48,2.67)	1.56 (0.79,3.30)^‡^	0.66 (0.38,1.14)	**0.0087**
Hematocrit (%, n = 212)	0.43 ± 0.05	0.41 ± 0.05	0.43 ± 0.06	0.42 ± 0.04	0.095
Hemoglobin (g/l, n = 212)	148 ± 18∗	139 ± 15^†^	150 ± 17	143 ± 14	**0.0087**
Creatinine (umol/l, n = 224)	95.8 ± 22.8^†‡^	93.0 ± 22.9^†‡^	81.4 ± 14.8	78.5 ± 13.8	**<0.0001**
GFR (ml/min/1.73m^2^, n = 224)	78 ± 20^†‡^	74 ± 22^†‡^	92 ± 14	90 ± 13	**<0.0001**
Blood glucose (mmol/l, n = 224)	6.2 ± 2.1	6.4 ± 2.2	6.2 ± 2.7	5.1 ± 0.6	0.092
NT-proBNP (pg/ml, n = 196)	325 (153,849)∗^†‡^	842 (258,2051)^†‡^	23 (16,36)	37 (17,84)	**<0.0001**
ACEI/ARB (%)	75.9^†‡^	64.0^†‡^	38.9^‡^	2.0	**<0.0001**
β-blocker (%)	71.3^†‡^	68.0^‡^	47.2^‡^	10.0	**<0.0001**
Ca^2+^ antagonists (%)	46.3^‡^	32.0^‡^	33.3^‡^	6.0	**<0.0001**
Statin (%)	37.0^‡^	34.0^‡^	27.8^‡^	4.0	**0.0002**
Aspirin (%)	22.2	24.0	19.4	6.0	0.070
Diuretic (%)	67.6^†‡^	56.0^†‡^	8.3	0	**<0.0001**

Values are given as mean ± SD or medians (interquartile range) or percentages. The P-values indicate the statistic power among all four group. Bold P-values indicate a significance level of <0.05.

∗p < 0.05 vs. patients with HFpEF and normal weight; ^†^p < 0.05 vs. patients with obesity; ^‡^p < 0.05 vs. controls using post hoc Bonferroni analysis.

HFpEF, heart failure with preserved ejection fraction; BP, blood pressure; PV, plasma volume; SAHS, obstructive sleep apnea hypopnea syndrome; Hs-CRP, hypersensitive-C reactive protein; GFR, glomerular filtration rate; NT-proBNP, N-terminal pro–brain natriuretic peptide; ACEI/ARB, Angiotensin-Converting Enzyme Inhibitors/Angiotensin Receptor Blockers.

### Cardiac remodeling and function derived from echocardiography and CMR

**Fig. 3:**
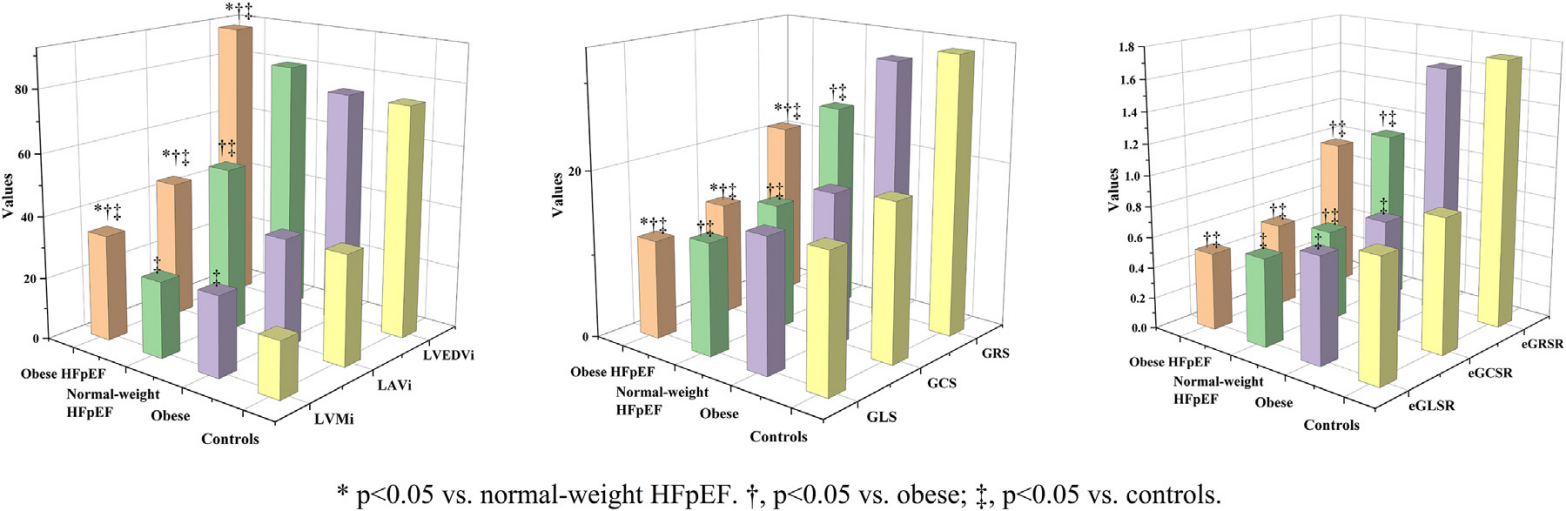
**Comparison of CMR parameters in four groups.** Data are presented as bars with mean. Strain parameters were expressed as absolute values, and the lower values meant worse cardiac function. Obese HFpEF, patients with HFpEF and obesity; normal weight HFpEF, patients with HFpEF and normal weight; obese, patients with obesity; LVMi, left ventricular end-diastolic mass/height^2.7^; EDVi, end-diastole volume index; LAVi, left atrial maximal volume index; GLS, GCS, and GRS, global longitudinal, circumferential, radial strain; eGLSR, eGCSR and eGRSR, early-diastolic GLS, GCS, GRS rate.

On echocardiography, patients with HFpEF and obesity showed no significant difference in LV mass index and relative LV wall thickness compared to patients with HFpEF and normal weight (Supplementary Table S1). CMR data are shown in Table 2 and Fig. 3. More severe LV remodeling parameters, including higher end-diastolic mass index (LVMi), end-diastolic volume index (LVEDVi) and end-systolic volume index (LVESVi), while lower LV end-diastolic diameter index (LVEDDi) and LA maximal volume index (LAVi) were observed in patients with HFpEF and obesity versus patients with HFpEF and normal weight. In comparison to patients with obesity and controls, patients with HFpEF and obesity had slightly lower LVEF (but still within the HFpEF range), yet higher LAVi, LVMi, LVEDVi, and LVESVi. Moreover, patients with obesity had higher LVMi, yet lower LVEDDi versus controls. After adjusting for age, atrial fibrillation, and coronary artery disease between the patients with HFpEF and obesity and patients with HFpEF and normal weight groups, the former only showed higher LAV (95 ± 3 vs.75 ± 5 ml, p = 0.0013) and LVEDD (56 ± 1 vs.51 ± 1 mm, p = 0.0003), but lower LVEDDi (27.7 ± 0.4 vs. 30.2 ± 0.6 mm/m^2^, p = 0.0003) in CMR imaging (Table 3).

**Table 2:** CMR data.

Variables	Patients with HFpEF and obesity (n = 108)	Patients with HFpEF and normal weight (n = 50)	Patients with Obesity (n = 72)	Controls (n = 50)	P-value
LVEF (%)	56 ± 6^†‡^	59 ± 7^‡^	60 ± 5	62 ± 4	**<0.0001**
Heart rate (bpm)	70 ± 12	69 ± 13	70 ± 12	69 ± 11	0.90
Cardiac index (ml/m^2^)	3.5 ± 0.8	3.2 ± 0.8	3.2 ± 0.8	3.2 ± 0.7	**0.023**
LAV (ml)	80 (67,109)^†‡^	84 (67,100)^†‡^	73 (58,85)^‡^	56 (47,68)	**<0.0001**
LAVi (ml/m^2^)	38.4 (31.9,52.4)∗^†‡^	49.2 (39.4,60.4)^†‡^	34.7 (29.7,41.0)	34.3 (28.0,40.7)	**<0.0001**
LVEDD (mm)	57 ± 6∗^†‡^	50 ± 6	51 ± 6^‡^	47 ± 4	**<0.0001**
LVEDDi (mm/m^2^)	27.7 ± 3.2∗^†^	30.3 ± 4.0^†^	24.8 ± 3.0^‡^	28.6 ± 2.3	**<0.0001**
LVEDVi (ml/m^2^)	91.0 ± 21.5∗^†‡^	81.0 ± 20.5	75.1 ± 13.4	74.9 ± 11.4	**<0.0001**
LVESVi (ml/m^2^)	40.6 ± 13.0∗^†‡^	34.1 ± 12.2	30.1 ± 7.6	28.7 ± 6.2	**<0.0001**
LVMi (g/m^2.7^)	34.1 ± 10.4∗^†‡^	24.6 ± 12.2^‡^	25.9 ± 5.3^‡^	18.1 ± 3.0	**<0.0001**
GLS (%)	−11.8 ± 3.0∗^†‡^	−13.4 ± 2.7^†‡^	−16.0 ± 2.8	−16.3 ± 2.1	**<0.0001**
GCS (%)	−13.5 ± 3.4∗^†‡^	−15.1 ± 3.1^†‡^	−18.2 ± 2.7	−18.9 ± 1.7	**<0.0001**
GRS (%)	20.9 ± 7.4∗^†‡^	24.7 ± 7.5^†‡^	31.5 ± 7.2	33.3 ± 4.8	**<0.0001**
sGLSR (/s)	−0.66 ± 0.16^†‡^	−0.71 ± 0.21^†‡^	−0.82 ± 0.13	−0.83 ± 0.15	**<0.0001**
sGCSR (/s)	−0.80 ± 0.21^†‡^	−0.82 ± 0.22^†‡^	−0.95 ± 0.14	−0.97 ± 0.16	**<0.0001**
sGRSR (/s)	1.19 ± 0.43^†‡^	1.29 ± 0.44^†‡^	1.61 ± 0.35	1.70 ± 0.37	**<0.0001**
eGLSR (/s)	0.50 ± 0.17^†‡^	0.57 ± 0.25^‡^	0.69 ± 0.17^‡^	0.79 ± 0.21	**<0.0001**
eGCSR (/s)	0.54 ± 0.20^†‡^	0.59 ± 0.28^†‡^	0.75 ± 0.20^‡^	0.87 ± 0.20	**<0.0001**
eGRSR (/s)	−0.97 ± 0.44^†‡^	−1.10 ± 0.50^†‡^	−1.61 ± 0.61	−1.72 ± 0.42	**<0.0001**
Presence of LGE (n = 140)	35.4 (29/82)	19.4 (6/31)	12.5 (3/24)	0 (0/3)	0.071
LGE percentages (%/LV)	1.0 ± 2.8	1.2 ± 3.8	0.3 ± 0.9	0	0.21
Native T1 (ms, n = 98)	1322 ± 103	1295 ± 168	1274 ± 81	–	0.26
ECV (%, n = 98)	30.6 ± 5.2^†^	30.3 ± 3.6^†^	26.5 ± 3.8	–	**0.0042**

Values are given as mean ± SD, or median (interquartile range), or n (%). The P-values indicate the statistic power among all four group.

Bold P-values indicate a significance level of <0.05.

∗p < 0.05 vs. patients with HFpEF and normal weight; ^†^p < 0.05 vs. patients with obesity; ^‡^p < 0.05 vs. controls using post hoc Bonferroni analysis.

CMR, cardiovascular magnetic resonance; LV, left ventricular; LA, left atrial; EF, ejection fraction; LVEDD/LVEDDi, LV end-diastolic diameter index; LVMi, LV end-diastolic mass/height^2.7^; EDVi/ESVi, end-diastole/systole volume index; LAV/LAVi, LA maximal volume index; GLS, GCS, and GRS, global longitudinal, circumferential, radial strain; sGLSR, sGCSR and sGRSR, systolic GLS, GCS, GRS rate; eGLSR, eGCSR and eGRSR, early-diastolic GLS, GCS, GRS rate; LGE, late gadolinium enhancement; ECV, extracellular volume fraction.

**Table 3:** Comparisons of CMR data in patients with HFpEF using analysis of covariance.

Variables	Patients with HFpEF and obesity (n = 108)	Patients with HFpEF and normal weight (n = 50)	P-value
LVEF (%)	56 ± 1	58 ± 1	0.35
Heart rate (bpm)	70 ± 1	69 ± 2	0.88
Cardiac index (ml/m^2^)	3.4 ± 0.1	3.4 ± 0.1	0.73
LAV (ml)	95 ± 3	75 ± 5	**0.0013**
LAVi (ml/m^2^)	47.5 ± 1.7	45.8 ± 2.6	0.62
LVEDD (mm)	56 ± 1	51 ± 1	**0.0003**
LVEDDi (mm/m^2^)	27.7 ± 0.4	30.2 ± 0.6	**0.0003**
LVEDVi (ml/m^2^)	87.6 ± 2.0	88.2 ± 3.2	0.88
LVESVi(kg/m^2^)	38.8 ± 1.2	38.1 ± 2.0	0.77
LVMi (g/m^2.7^)	32.2 ± 1.0	28.8 ± 1.5	0.089
GLS (%)	−11.9 ± 0.3	−13.2 ± 0.5	**0.031**
GCS (%)	−13.7 ± 0.3	−14.7 ± 0.5	0.15
GRS (%)	21.5 ± 0.7	23.3 ± 1.2	0.22
sGLSR (/s)	−0.67 ± 0.02	−0.70 ± 0.03	0.35
sGCSR (/s)	−0.79 ± 0.02	−0.83 ± 0.03	0.46
sGRSR (/s)	1.20 ± 0.04	1.26 ± 0.07	0.49
eGLSR (/s)	0.49 ± 0.02	0.57 ± 0.03	**0.043**
eGCSR (/s)	0.54 ± 0.02	0.59 ± 0.04	0.34
eGRSR (/s)	−0.97 ± 0.05	−1.09 ± 0.07	0.23
ECV (%, n = 79)	30.7 ± 0.7	30.0 ± 1.2	0.64

Adjustment of confounding baseline factors, including age, atrial fibrillation, coronary artery disease in obesity or normal weight patients with HFpEF using analysis of covariance.

Values are given as mean ± SE.

Bold P-values indicate a significance level of <0.05.

CMR, cardiovascular magnetic resonance; LV, left ventricular; LA, left atrial; LVEDD/LVEDDi, LV end-diastolic diameter index; LVMi, LV end-diastolic mass/height^2.7^; EDVi/ESVi, end-diastole/systole volume index; LAV/LAVi, LA maximal volume index; GLS, GCS, and GRS, global longitudinal, circumferential, radial strain; sGLSR, sGCSR and sGRSR, systolic GLS, GCS, GRS rate; eGLSR, eGCSR and eGRSR, early-diastolic GLS, GCS, GRS rate; ECV, extracellular volume fraction.

### CMR derived myocardial strain and correlates with echocardiography

**Fig. 4:**
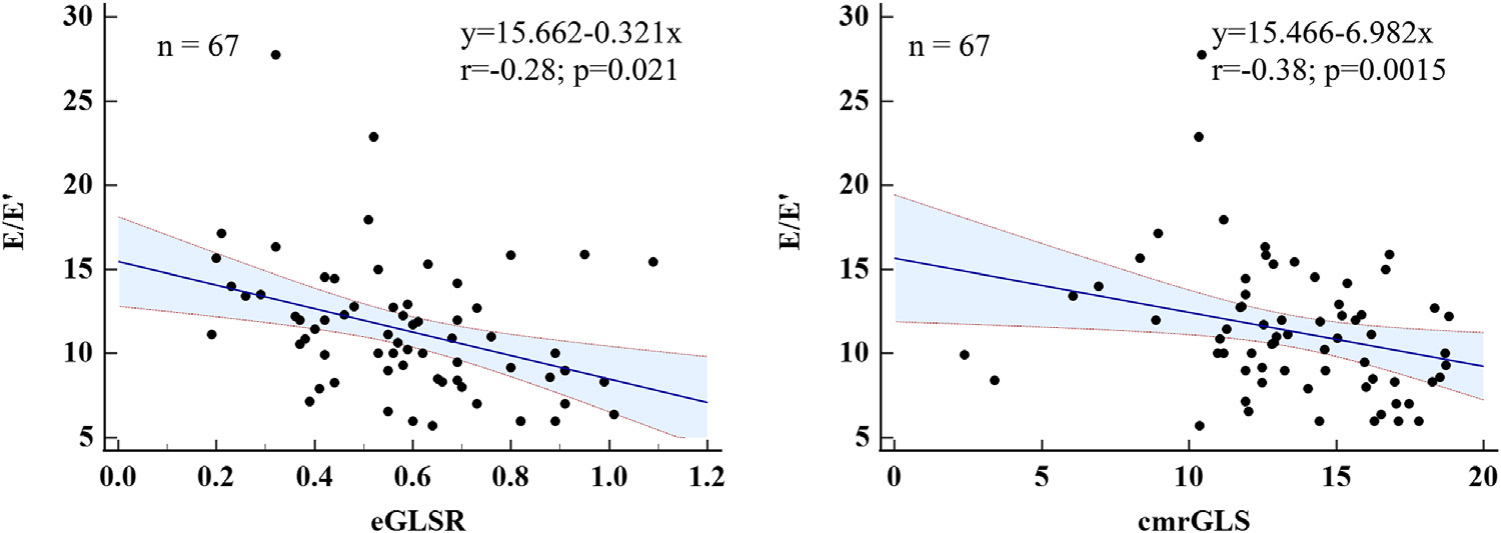
**Scatterplot showing a mild correlation between E/E′ and GLS and early-diastolic longitudinal strain rate in all patients.** Strain parameters were expressed as absolute values, and the lower values meant worse cardiac function. The blue shaded regions represented the 95% prediction intervals. E, early-diastolic mitral inflow velocity; E′, mean mitral annular peak early-diastolic velocity; GLS, global longitudinal strain; eGLSR, early-diastolic GLS rate.

With regards to myocardial strain, patients with HFpEF and obesity had more severe LV dysfunction— with expectedly worse GLS, GCS, and GRS versus patients with HFpEF and normal weight (all p < 0.05). Compared to patients with obesity and controls, patients with HFpEF both had significantly worse strains and strain rates (p < 0.05). In addition, patients with obesity also showed worse eGLSR and eGCSR compared with controls. Overall comparisons of strain parameters amongst the four groups were plotted in Fig. 3 and supplementary Fig. S1. After adjusting for age, atrial fibrillation, and coronary artery disease, GLS (−11.9 ± 0.3% vs. −13.2 ± 0.5%, p = 0.031), and eGLSR (0.49 ± 0.02 vs. 0.57 ± 0.03/s, p = 0.043) were still appreciably worse in patients with HFpEF and obesity versus patients with HFpEF and normal weight (Table 3). In HFpEF patients without coronary artery disease or atrial fibrillation subgroup (Supplementary Tables S2−S5), majority of parameters kept the same significant differences with the overall HFpEF spectrum, while in HFpEF patients without atrial fibrillation subgroup (Supplementary Table S3), significant differences disappeared in GLS between patients with HFpEF and obesity or normal weight. Moreover, GLS showed moderate levels of inter-modality agreement (r = 0.57, p < 0.0001) between speckle tracking and CMR-FT (Fig. S2); early-diastolic mitral inflow velocity/mean mitral annular peak early-diastolic velocity (E/E’) had a mild correlation with CMR derived GLS (r = −0.28, p = 0.021) and eGLSR (r = −0.38, p = 0.0015) in all patients (Fig. 4).

### Myocardial tissue characteristics derived from CMR LGE and T1 mapping

Of the 280 patients, 140 (50%) patients completed contrast enhanced CMR examination. Among them, 35.4% (29/82) patients with HFpEF and obesity, 19.4% (6/31) patients with HFpEF and normal weight, 12.5% (3/24) patients with obesity and no control participant showed positive LGE (Table 2). No remarkable difference was observed of quantitative LGE among four groups. In addition, 35% (98/280) patients completed T1 mapping examination, and patients with HFpEF showed significantly elevated ECV—myocardial interstitial fibrosis compared to patients with obesity. However, patients with HFpEF and obesity showed no significant difference of native T1 and ECV values compared with patients with HFpEF and normal weight. There was also no difference of ECV between patients with obesity and normal weight HFpEF groups independent of age, atrial fibrillation, and coronary artery disease (Supplementary Tables S2–S5).

### Correlates of myocardial strain with tissue characteristics

We further divided 82 patients with HFpEF and obesity underwent delayed-enhancement CMR examination into with (n = 53) or without LGE (n = 29) groups, and Table 4 summarized their mean strain values. Patients with HFpEF and obesity with LGE had worse GRS, eGLSR, eGCSR, and eGRSR than patients without LGE. In addition, GCS, eGLSR, eGCSR, and eGRSR showed significant association with the presence of LGE both in patients with HFpEF and obesity (ρ = 0.24–0.35, p < 0.05) and all enrolled patients (ρ = 0.26–0.36, p < 0.05). However, there was no significant association between ECV and early-diastolic strain rates or E/E’ value in patients with HFpEF and obesity and all enrolled cohort (all p > 0.05, Supplementary Tables S6 and S7). Moreover, GLS, GCS, and GRS all showed better diagnostic ability (area of curves, AUC = 0.71–0.73, p < 0.005) than ECV to diagnose patients with HFpEF and obesity from patients with HFpEF and normal weight. Especially, GLS showed highest diagnostic ability (AUC = 0.912, p < 0.0001) than traditional CMR parameters and ECV to diagnose patients with HFpEF and obesity in the setting of obesity (Table 5).

**Table 4:** Comparison of CMR derived strain values in patients with HFpEF and obesity without and with LGE.

Variables	LGE (−) (n = 53)	LGE (+) (n = 29)	P-value
GLS (%)	−11.9 ± 3.2	−11.0 ± 2.5	0.20
GCS (%)	−13.6 ± 3.7	−12.2 ± 2.4	0.053
GRS (%)	21.2 ± 7.7	17.9 ± 4.5	**0.017**
sGLSR (/s)	−0.67 ± 0.16	−0.62 ± 0.13	0.19
sGCSR (/s)	−0.80 ± 0.25	−0.74 ± 0.16	0.20
sGRSR (/s)	1.19 ± 0.46	1.03 ± 0.27	0.089
eGLSR (/s)	0.52 ± 0.17	0.44 ± 0.14	**0.033**
eGCSR (/s)	0.57 ± 0.21	0.42 ± 0.13	**0.0003**
eGRSR (/s)	−0.99 ± 0.43	−0.76 ± 0.32	**0.0063**
ECV (%)	30.3 ± 4.9	31.2 ± 5.7	0.52

Data presented as mean ± SD.

Bold P-values indicate a significance level of <0.05.

GLS, GCS, and GRS, global longitudinal, circumferential, radial strain; sGLSR, sGCSR and sGRSR, systolic GLS, GCS, GRS rate; eGLSR, eGCSR and eGRSR, early-diastolic GLS, GCS, GRS rate; ECV, extracellular volume fraction.

**Table 5:** Identifying patients with HFpEF and obesity in overall HFpEF spectrum and patients with obesity.

Variables	Differentiate patients with HFpEF and obesity from overall patients with HFpEF (n = 79)			Differentiate with HFpEF and obesity from patients with obesity (n = 77)		
	AUC	95%CI	p-value	AUC	95%CI	p-value
LVEF	0.656	0.516–0.797	**0.035**	0.773	0.650–0.896	**0.0004**
LAVi	0.649	0.512–0.786	**0.045**	0.615	0.485–0.744	0.14
LVEDVi	0.652	0.517–0.788	**0.040**	0.736	0.617–0.855	**0.0021**
LVESVi	0.686	0.550–0.822	**0.012**	0.783	0.671–0.895	**0.0002**
LVMi	0.836	0.731–0.940	**<0.0001**	0.711	0.599–0.824	**0.0059**
GLS	0.709	0.582–0.837	**0.005**	0.912	0.833–0.991	**<0.0001**
GCS	0.722	0.600–0.843	**0.0027**	0.890	0.802–0.979	**<0.0001**
GRS	0.733	0.614–0.852	**0.0017**	0.878	0.786–0.971	**<0.0001**
eGLSR	0.554	0.406–0.702	0.46	0.749	0.636–0.863	**0.0012**
ECV	0.526	0.391–0.661	0.73	0.746	0.616–0.875	**0.0014**

Bold P-values indicate a significance level of <0.05.

AUC, area under the ROC curves; CI, confidence interval; LV, left ventricular; EDVi/ESVi, end-diastole/systole volume index; LVMi, LV end-diastolic mass/height^2.7^; LAVi, left atrial maximal volume index; GLS, GCS, and GRS, global longitudinal, circumferential, radial strain; eGLSR, early-diastolic GLS rate; ECV, extracellular volume fraction.

### Data reproducibility

The CoV, ICC, and Bland–Altman Plots for strain measurements are shown in supplementary Table S8 and Fig. S3. Global LV strain and strain rate were reproducible at the intra- and interobserver level, and all showed excellent ICC (>0.93) and CoV less than 15%.

## Discussion

**Fig. 5:**
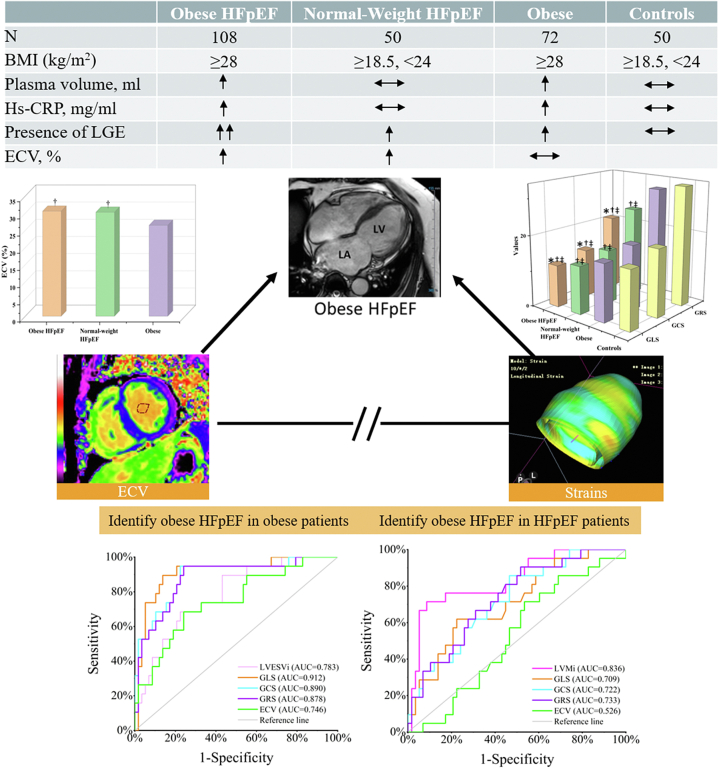
**Clinical and CMR features in patients with HFpEF and obesity, patients with HFpEF and normal weight, patients with obesity, and control cohorts.** Strains showed potential to identify patients with HFpEF and obesity from all patients with HFpEF and patients with obesity (the bottom line). Obese HFpEF, patients with HFpEF and obesity; normal weight HFpEF, patients with HFpEF and normal weight; obese, patients with obesity; BMI, body mass index; CRP, hypersensitive-C reactive protein; LGE, late gadolinium enhancement; ECV, extracellular volume fraction; GLS, GCS, and GRS, global longitudinal, circumferential, radial strain; LVMi, left ventricular end-diastolic mass/height^2.7^; ESVi, end-systole volume index; AUC, area under the curve.

The present study innovatively provides detailed clinical and laboratory phenotyping along with CMR strain and tissue characteristics for the poorly described population of patients with HFpEF and obesity, compared with patients with HFpEF and normal weight, patients with obesity, and control individuals. The major findings were: (i) compared with patients with HFpEF and normal weight, patients with HFpEF and obesity phenotype were younger, showed higher systemic inflammation, less commonly atrial fibrillation, more severe LV remodeling and cardiac dysfunction, but similar fibrosis; (ii) GLS and eGLSR outperformed structural parameters in illustrating the abnormalities of patients with HFpEF and obesity independent of age, atrial fibrillation, and coronary artery disease; (iii) strains and ECV may represent different mechanisms of patients with HFpEF and obesity; (iv) GLS may better diagnose patients with HFpEF and obesity in overall patients with obesity (Fig. 5).

Obesity is a commonly occurring comorbidity in patients with HFpEF,^6^ and has many deleterious effects on the cardiovascular system, mediated by changes in volume overload, cardiac load, energy substrate utilization, tissue metabolism, and systemic inflammation.^2^^,^^21^ Evidence has suggested that patients with HFpEF and comorbid obesity may represent a clinically relevant, distinct phenotype within the broad spectrum of HFpEF individuals.^1^^,^^22^ Our data are congruent with previous publications.^7^ Despite the similar NYHA class, patients with HFpEF and obesity demonstrated higher uric acid levels versus patients with HFpEF and normal weight and controls, indicating more severe systemic inflammation. Moreover, the lower NT-proBNP levels may relate to increased adipose tissue and higher plasma volume levels in patients with HFpEF and obesity.^23^^,^^24^ However, the obvious volume overload in patients with HFpEF and obesity was not proved, which may be attributable to no direct parameters of LV filling pressures from catheters in our study. Our study also expanded the previous study,^2^^,^^7^ showing the prototypal obese HFpEF phenotype—representative of the Chinese population, which were roughly two decade younger compared with patients with HFpEF and normal weight group. This may help understand the actual distribution of the population with obesity—the young and middle-aged males in China,^25^^,^^26^ and put a heavier burden in medical management.

The present data also confirmed and extended previous studies,^2^^,^^7^ showing that patients with HFpEF and obesity had worse cardiac remodeling—higher LVMi, LVEDVi, LVESVi, while relatively lower LAVi, when compared with patients with HFpEF and normal weight by the Chinese obesity criteria. Besides, part of cardiac remodeling was also observed in patients with obesity versus controls, both indicating the distinct role of obesity. However, the difference of LVMi, LVEDVi, and LAVi disappeared in obesity and normal weight patients with HFpEF after adjustment for age, atrial fibrillation, coronary artery disease and sensitivity analysis, and therefore more sensitive parameters may be required to better identify patients with HFpEF and obesity from overall patients with HFpEF.

A significant gap remains about the subclinical cardiac dysfunction in patients with HFpEF and obesity when compared to their normal weight HFpEF counterparts, despite the preserved LVEF. Complementary to LVEF, myocardial strain provides the added benefit of not only allowing the assessment of global systolic function, but of diastolic function.^27^ Furthermore, the development of CMR-FT has enabled a highly precise evaluation of myocardial strain without the necessity of additional image acquisition.^9^^,^^11^ In addition to cardiac remodeling, we found for the first time that strain parameters (GLS, GCS, and GRS) were all further impaired in comparison to the patients with HFpEF and normal weight group, reflecting the severe subclinical cardiac dysfunction in patients with HFpEF and obesity. This can also be observed in the comparison between patients with obesity and controls, as the former group also showed diastolic dysfunction, demonstrated by impaired eGLSR and eGCSR. Of note, as emphasized by the present data, GLS and eGLSR—correlated with echocardiographic diastolic dysfunction (E/E’), were significantly worse in the patients with HFpEF and obesity when compared to patients with HFpEF and normal weight independent of age, atrial fibrillation, and coronary artery disease. Our study further expanded the use of GLS and eGLSR as a parameter to differentiate and confirm severe diastolic dysfunction in the patients with HFpEF and obesity phenotype rather than traditional parameters, which may indicate the progressive myocardial fiber impairment in patients with HFpEF and obesity.^11^

For tissue characteristics, though elevated ECV was observed in overall HFpEF spectrum, no significant difference of LGE presence and ECV was observed between obesity and normal weight patients with HFpEF, indicating similar diffuse fibrosis in HFpEF patients. To note, previous publications reported that GLS and eGLSR minimally correlated with ECV in 100–1000 enrolled patients,^28^^,^^29^ while in our study, we found that early-diastolic strain rates mildly correlated with the LGE presence, rather than ECV in patients with HFpEF and obesity (n = 58). As the GCS and GRS were significantly correlated with ECV values in all enrolled patients (n = 98), we presumed that with the increased sample size with ECV, the correlation between GLS and ECV may appear. However, as the correlation between strains and ECV was minimal, this may represent different mechanisms of strain and ECV in patients with HFpEF and obesity, and therefore one was not a proxy for the other, deserving further investigation.

In addition, as challenges still existed in diagnosing the HFpEF in the setting of patients with obesity for a variety of reasons, including lower NT-proBNP levels, and the overlap of symptoms,^5^ our study possibly provided new noninvasive GLS and eGLSR parameters. These finding supported the application of strain parameters for assessing clinical presentation and may become an accessible and supplemental parameter in patients with HFpEF and obesity monitoring and early diagnosis. Nonetheless, we have now merely embarked on a potentially revolutionary journey of the application of CMR-FT in the obese HFpEF phenotype, and more research is needed to establish the role of CMR-FT in routine clinical decision-making processes.

There are some limitations in our study. Firstly, relatively small samples performed in a single-centre limited the generalizability, therefore subtle associations may not have been detectable, especially in sensitivity analysis with limited sample size and some CMR parameters (ECV). However, this study represented a preliminary analysis, a large-scale prospective study is now being conducted to further confirm the results and ascertain prognostic value. Secondly, the application of the HFA-PEFF score as implemented within the study excluded patients with intermediate scores, who were recommended to undergo stress tests (e.g. right heart catheterization) to diagnose or exclude the presence of HFpEF. Therefore, the given conclusions might be prone to a selection bias. However, the invasiveness of catheterization limits its clinical generalizability, we comprehensively considered the existing accurate and accessible diagnostic methods from guidelines and previous literatures to diagnose HFpEF,^30^^,^^31^ which may also affect the conclusion like the association between plasma volume load and LV filling pressure. Thirdly, the baseline data of patients with obesity and controls were not fully matched, which may confound the real effect of obesity in cardiac function. As the consecutive enrolment essence of this study, we avoid selection bias to the greatest extent. Finally, not all echo tissue doppler imaging was available, since we comprehensively considered present parameters to diagnose HFpEF according to the latest guidelines.^15^

In this well-defined cohort of consecutively studied heart failure patients, the obesity-related HFpEF phenotype showed more obvious systemic inflammation, and prominent impaired GLS and eGLSR, assisting in the promising strain-tailored management of patients with HFpEF and obesity phenotype; strains and ECV may represent different mechanisms of obese HFpEF. Our findings provide a basis for an improved identification algorithm and personalized therapy of patients with HFpEF and obesity using comprehensive CMR approach addressing strain parameters. Further large-scale investigations augmenting the prognostic value of strain and ECV, and their mechanisms are necessary.

## Contributors

ML conceived the study and designed the protocols; JH, WY, SL, BZ, GY and JX collected the data; WW reviewed the echocardiographic data; JH, WY, DZ, LZ and YW conducted the analysis and interpreted the results; JH drafted the first version of the manuscript; JH and WY accessed and verified the underlying data; XS, YZ, PS, FK, SZ, ZT and AS made critical revisions of the manuscript; ML obtained the project funding; ML and SZ takes responsibility for the integrity of the data and the accuracy of the data analysis.

## Data sharing statement

Data are available from the corresponding author (ML) upon reasonable request for research purposes once the data have been de-identified and all the main findings have been published.

## Declaration of interests

JH reports grants from the Construction Research Project of Key Laboratory (Cultivation) of 10.13039/501100005150Chinese Academy of Medical Sciences (2019PT310025); the 10.13039/501100001809National Natural Science Foundation of China (81971588); the Capital's Funds for Health Improvement and Research (CFH 2020-2-4034); the Youth Key Program of High-level Hospital Clinical Research (2022-GSP-QZ-5), and CAMS Innovation Fund for Medical Sciences (CIFMS, 2022-I2M-C&T-B-052).
