# Supplementary material for: Clinical features, myocardial strain and tissue characteristics of heart failure with preserved ejection fraction in patients with obesity: A prospective cohort study
Source: eClinicalMedicine. 2022 Nov 3;55:101723. doi: 10.1016/j.eclinm.2022.101723 (PMC9646878; doi:10.1016/j.eclinm.2022.101723)
Supplement: Multimedia component 1 [file mmc1.docx]

**Supplementary Files**

Table S1. Echocardiography data

| **Variables** | **Patients with HFpEF and obesity**  **(n=108)** | **Patients with HFpEF and normal weight**  **(n=50)** | **Patients with obesity**  **(n=72)** | **Controls**  **(n=50)** | **P-value** |
| --- | --- | --- | --- | --- | --- |
| LVEF (%) | 55±8*^†‡^ | 58±8^†‡^ | 64±4 | 65±4 | **<0·0001** |
| E (cm/s, n=276) | 80±22 | 86±21 | 81±21 | 89±16 | **0·047** |
| E’, septal (cm/s, n=65) | 6·2±1·8^‡^ | 6·3±2·4^‡^ | 8·4±2·3 | 10·6±2·7 | **<0·0001** |
| E’, lateral (cm/s, n=65) | 7·8±2·4^‡^ | 8·3±3·3^‡^ | 11·2±2·5 | 12·3±2·9 | **<0·0001** |
| E/E’, septal (n=65) | 13·5±4·8^‡^ | 14·6±4·5^‡^ | 9·9±2·7 | 8·8±2·4 | **0·0007** |
| E/E’, lateral (n=65) | 10·8±3·6^‡^ | 11·7±5·1^‡^ | 7·3±1·7 | 7·5±2·0 | **0·0082** |
| E/E’, average (n=67) | 11·8±3·3^‡^ | 13·1±4·7^‡^ | 7·2±1·3 | 8·2±2·3 | **0·0014** |
| LV mass index (g/m^2^) | 122·7±33·6^†‡^ | 115·6±47·8^†‡^ | 88·0±16·7 | 83·0±21·6 | **<0·0001** |
| RWT | 0·38±0·08 | 0·42±0·15 | 0·39±0·06 | 0·37±0·06 | 0·063 |
| GLS (%, n=44) | -15·1±3·4^‡^ (n=28) | -15·6±3·7 (n=11) | - | -20·2±3·6 (n=5) | **0·017** |
| LADap (mm) | 42·5±5·6^†‡^ | 42·0±5·5^†‡^ | 36·7±3·7^‡^ | 32·4±2·8 | **<0·0001** |

Values are given as mean ± SD or n (%). The P-values indicate the statistic power among all four groups. Bold P-values indicate a significance level of <0.05. * p<0.05 vs. patients with HFpEF and normal weight; †, p<0.05 vs. patients with obesity; ‡, p<0.05 vs. controls using post hoc Bonferroni analysis. LV, left ventricular; EF, ejection fraction; E, early-diastolic mitral inflow velocity; E’, mean mitral annular peak early diastolic velocity; RWT, relative wall thickness; GLS, global longitudinal strain; LADap, anteroposterior left atrial diameter.

Table S2 Baseline and CMR data in patients without atrial fibrillation

| **Variables** | **Patients with HFpEF and obesity（n=81）** | **Patients with HFpEF and normal weight**  **(n=25)** | **Patients with obesity**  **（n=70）** | **Controls**  **(n=50)** | **P-value** |
| --- | --- | --- | --- | --- | --- |
| **Baseline data** |  |  |  |  |  |
| Age (years) | 43±14* | 65±9^†‡^ | 46±12 | 44±10 | **<0·0001** |
| Male (%) | 75·3^‡^ | 56·0 | 74·3^‡^ | 44·0 | **<0·0007** |
| Female (%) | 24**·**7^‡^ | 44·0 | 25·7^‡^ | 56·0 | **<0·0007** |
| Body mass index (kg/m^2^) | 31±2*^‡^ | 22±2^†^ | 31±2^‡^ | 22±1 | **<0·0001** |
| NYHA class III/IV (%) | 37·0 | 32·0 | 0 | 0 | 0·30 |
| Systolic BP (mmHg, n=206) | 138±24^‡^ | 137±21 | 139±16^‡^ | 124±15 | **0·0039** |
| Diastolic BP (mmHg, n=206) | 87±17^‡^ | 79±12 | 85±11^‡^ | 77±10 | **0·0019** |
| Estimated PV (ml, n=164) | 2968±322*^‡^ | 2355±325^†^ | 2940±336^‡^ | 2301±191 | **<0·0001** |
| Diabetes (%) | 32·1^‡^ | 40·0^‡^ | 25·7^‡^ | 4·0 | **0·0007** |
| Hypertension (%) | 98·8^†‡^ | 92·0^‡^ | 75·7^‡^ | 14·0 | **<0·0001** |
| Coronary artery disease (%) | 11·1* | 40·0^†‡^ | 4·3 | 0 | **<0·0001** |
| SAHS (%) | 14·8^‡^ | 0 | 22·9^‡^ | 0 | **<0·0001** |
| Dyslipidemia (%) | 46·9 | 64·0^‡^ | 52·9^‡^ | 26·0 | **0·0054** |
| Uric acid(μmol/l, n=171) | 454·0±137·2^†‡^ | 384·1±107·4 | 383·3±83·5 | 326·9±70·6 | **<0·0001** |
| HsCRP(mg/l, n=167) | 1·49(0·83,4·36)^‡^ | 1·44(0·55,3·34) | 1·70(0·79,3·32)^‡^ | 0·66(0·38,1·14) | **0·0092** |
| Hematocrit (%, n=164) | 0·44±0·05 | 0·41±0·05 | 0·43±0·06 | 0·42±0·04 | 0·14 |
| Hemoglobin(g/l, n=164) | 148±18 | 138±15^†^ | 150±17 | 143±14 | **0·025** |
| Creatinine(umol/l, n=172) | 96·7±23·8^†‡^ | 99·0±27·2^†‡^ | 81·9±14·7 | 78·5±13·8 | **<0·0001** |
| GFR(ml/min/1·73m^2^, n=172) | 79±21^†^ | 71±21^†‡^ | 91±14 | 90±13 | **<0·0001** |
| Blood glucose(mmol/l, n=172) | 6·4±2·3 | 7·0±2·9^‡^ | 6·2±2·7 | 5·1±0·6 | 0·045 |
| NT-proBNP (pg/ml, n=146) | 228(131,632)^†‡^ | 423(233,1846)^†‡^ | 23(16,35) | 37(17,84) | **<0·0001** |
| ACEI/ARB (%) | 77·8^†‡^ | 64·0^‡^ | 40·0^‡^ | 2·0 | **<0·0001** |
| β-blocker (%) | 70·4^†‡^ | 64·0^‡^ | 45·7^‡^ | 10·0 | **<0·0001** |
| Ca^2+^ antagonists (%) | 49·4^‡^ | 40·0 | 34·3^‡^ | 6·0 | **<0·0001** |
| Statin (%) | 38·3^‡^ | 48·0^‡^ | 28·6^‡^ | 4·0 | **<0·0001** |
| Aspirin (%) | 25·9^‡^ | 36·0^‡^ | 20·0 | 6·0 | **0·0094** |
| Diuretic (%) | 67·9^†‡^ | 56·0^†‡^ | 8·6 | 0 | **<0·0001** |
| **CMR data** |  |  |  |  |  |
| LVEF (%) | 56±6^†‡^ | 59±6 | 60±5 | 62±4 | **<0·0001** |
| Heart rate (bpm) | 69±12 | 69±15 | 70±12 | 69±11 | 0·88 |
| Cardiac index (ml/m^2^) | 3·5±0·8^†^ | 3·4±0·8 | 3·2±0·7 | 3·2±0·7 | **0·0089** |
| LAV (ml) | 79±22^‡^ | 78±39^‡^ | 72±16^‡^ | 58±14 | **<0·0001** |
| LAVi (ml/m^2^) | 38·8±10·9* | 47·6±24·7^†‡^ | 35·0±7·2 | 35·6±9·4 | **<0·0001** |
| LVEDD (mm) | 58±6*^†‡^ | 50±6 | 51±6^‡^ | 47±4 | **<0·0001** |
| LVEDDi (mm/m^2^) | 28·0±3·1*^†^ | 30·6±4·5^†^ | 24·9±3·0^‡^ | 28·6±2·3 | **<0·0001** |
| LVEDVi (ml/m^2^) | 94·6±21·4^†‡^ | 84·9±23·4 | 75·2±13·6 | 74·9±11·4 | **<0·0001** |
| LVESVi (ml/m^2^) | 42·3±13·6*^†‡^ | 35·6±13·8 | 30·2±7·7 | 28·7±6·2 | **<0·0001** |
| LVMi(g/m^2.7^) | 37·0±10·0*^†‡^ | 28·9±15·9^‡^ | 25·9±5·4^‡^ | 18·1±3·0 | **<0·0001** |
| GLS (%) | -12·0±2·7^†‡^ | -13·1±3·2^†‡^ | -16·0±2·7 | -16·3±2·1 | **<0·0001** |
| GCS (%) | -13·8±3·1*^†‡^ | -15·7±3·4^†‡^ | -18·2±2·8 | -18·9±1·7 | **<0·0001** |
| GRS (%) | 21·4±7·0*^†‡^ | 26·3±7·0^†‡^ | 31·4±7·3 | 33·3±4·8 | **<0·0001** |
| sGLSR (/s) | -0·66±0·15^†‡^ | -0·73±0·22^‡^ | -0·82±0·13 | -0·83±0·15 | **<0·0001** |
| sGCSR (/s) | -0·80±0·17^†‡^ | -0·87±0·24 | -0·97±0·14 | -0·97±0·16 | **<0·0001** |
| sGRSR (/s) | 1·20±0·39^†‡^ | 1·40±0·48^‡^ | 1·60±0·36 | 1·70±0·37 | **<0·0001** |
| eGLSR (/s) | 0·48±0·15^†‡^ | 0·49±0·22^†‡^ | 0·69±0·17^‡^ | 0·79±0·21 | **<0·0001** |
| eGCSR (/s) | 0·52±0·19^†‡^ | 0·53±0·22^†‡^ | 0·74±0·20^‡^ | 0·87±0·20 | **<0·0001** |
| eGRSR (/s) | -0·97±0·44^†‡^ | -1·12±0·49^†‡^ | -1·60±0·61 | -1·72±0·42 | **<0·0001** |
| Presence of LGE (n=105) | 38·3 (23/60) | 21·1 (4/19) | 13·0 (3/23) | 0 (0/3) | 0·15 |
| LGE percentages (%/LV) | 1·2±3·2 | 1·7±4·8 | 0·3±0·9 | 0 | 0·49 |
| Native T1 (ms, n=72) | 1309±101 | 1301±210 | 1272±82 | - | 0·56 |
| ECV (%, n=72) | 30·3±5·3^†^ | 29·4±2·5 | 26·6±4·8 | - | **0·019** |

Values are given as mean ± SD or percentages. The P-values indicate the statistic power among all four groups. Bold P-values indicate a significance level of <0·05. * p<0·05 vs. patients with HFpEF and normal weight; †, p<0·05 vs. patients with obesity; ‡, p<0·05 vs. controls using post hoc Bonferroni analysis. HFpEF, heart failure with preserved ejection fraction; BP, blood pressure; PV, plasma volume; SAHS, obstructive sleep apnea hypopnea syndrome; Hs-CRP, hypersensitive-C reactive protein; GFR, glomerular filtration rate; NT-proBNP, N-terminal pro–brain natriuretic peptide; ACEI/ARB, Angiotensin-Converting Enzyme Inhibitors/Angiotensin Receptor Blockers. CMR, cardiovascular magnetic resonance; LV, left ventricular; LA, left atrial; EF, ejection fraction; LVEDD, LV end-diastolic diameter, LVEDDi, LVEDD index; EDVi/ESVi, end-diastole/systole volume index; LVMi, LV end-diastolic mass/height^2.7^; LAV, LA maximal volume; LAVi, LAV index; GLS, GCS, and GRS, global longitudinal, circumferential, radial strain; sGLSR, sGCSR and sGRSR, systolic GLS, GCS, GRS rate; eGLSR, eGCSR and eGRSR, early-diastolic GLS, GCS, GRS rate; LGE, late gadolinium enhancement; ECV, extracellular volume fraction.

Table S3 Comparisons of CMR data in HFpEF patients without atrial fibrillation using analysis of covariance

| **Variables** | **Patients with HFpEF and obesity**  **（n=81）** | **Patients with HFpEF and normal weight**  **(n=25)** | **P-value** |
| --- | --- | --- | --- |
| LVEF (%) | 56±1 | 58±1 | 0·43 |
| Heart rate (bpm) | 68±1 | 72±3 | 0·29 |
| Cardiac index (ml/m^2^) | 3·4±0·1 | 3·8±0·2 | 0·12 |
| LAV (ml) | 81±3 | 72±6 | 0·24 |
| LAVi (ml/m^2^) | 40·4±1·8 | 42·5±3·5 | 0·61 |
| LVEDD (mm) | 57±1 | 52±1 | **0·012** |
| LVEDDi (mm/m^2^) | 28·1±0·4 | 30·1±0·8 | **0·029** |
| LVEDVi (ml/m^2^) | 91·7±2·5 | 94·4±5·0 | 0·65 |
| LVESVi(kg/m^2^) | 40·7±1·6 | 40·7±3·1 | 0·98 |
| LVMi(g/m^2.7^) | 35·8±1·3 | 32·7±2·6 | 0·33 |
| GLS (%) | -12·1±0·3 | -12·8±0·6 | 0·36 |
| GCS (%) | -14·2±0·4 | -14·6±0·7 | 0·65 |
| GRS (%) | 22·3±0·8 | 23·4±1·7 | 0·86 |
| sGLSR (/s) | -0·67±0·02 | -0·73±0·04 | 0·18 |
| sGCSR (/s) | -0·80±0·02 | -0·88±0·05 | 0·15 |
| sGRSR (/s) | 1·22±0·05 | 1·35±0·10 | 0·28 |
| eGLSR (/s) | 0·46±0·02 | 0·56±0·04 | **0·040** |
| eGCSR (/s) | 0·52±0·02 | 0·55±0·05 | 0·65 |
| eGRSR (/s) | -0·97±0·05 | -1·11±0·11 | 0·29 |
| ECV (%, n=72) | 30·6±0·8 | 28·4±1·6 | 0·25 |

Adjustment of confounding baseline factors, including age, coronary artery disease in patients with HFpEF and obesity and patients with HFpEF and normal weight using analysis of covariance. Values are given as mean ± SE. Bold P-values indicate a significance level of <0·05. HFpEF, heart failure with preserved ejection fraction; CMR, cardiovascular magnetic resonance; LV, left ventricular; LA, left atrial; EF, ejection fraction; LVEDD, LV end-diastolic diameter, LVEDDi, LVEDD index; EDVi/ESVi, end-diastole/systole volume index; LVMi, LV end-diastolic mass/height^2.7^; LAV, LA maximal volume; LAVi, LAV index; GLS, GCS, and GRS, global longitudinal, circumferential, radial strain; sGLSR, sGCSR and sGRSR, systolic GLS, GCS, GRS rate; eGLSR, eGCSR and eGRSR, early-diastolic GLS, GCS, GRS rate; LGE, late gadolinium enhancement; ECV, extracellular volume fraction.

| **Variables** | **Patients with HFpEF and obesity**  **（n=94）** | **Patients with HFpEF and normal weight**  **(n=37)** | **Patients with obesity**  **（n=69）** | **Controls**  **(n=50)** | **P-value** |
| --- | --- | --- | --- | --- | --- |
| **Baseline data** |  |  |  |  |  |
| Age (years) | 45±14* | 63±10^†‡^ | 45±11 | 44±10 | **<0·0001** |
| Male (%) | 73·4^‡^ | 51·4 | 73·9^‡^ | 44·0 | **0·0005** |
| Female (%) | 26·6^‡^ | 48·6 | 26·1^‡^ | 56·0 | **0·0005** |
| Body mass index (kg/m^2^) | 31±2*^‡^ | 22±2^†^ | 31±3^‡^ | 22±1 | **<0·0001** |
| NYHA class III/IV (%) | 41·5 | 29·7 | 0 | 0 | 0·10 |
| Systolic BP (mmHg, n=231) | 135±24 | 132±21 | 140±17^‡^ | 124±15 | **0·0034** |
| Diastolic BP (mmHg, n=231) | 85±16^‡^ | 79±13 | 85±11^‡^ | 77±10 | **0·0026** |
| Estimated PV (ml, n=190) | 2959±313*^‡^ | 2252±317^†^ | 2961±371^‡^ | 2302±191 | **<0·0001** |
| Atrial fibrillation (%) | 23·4*^†‡^ | 59·5^†‡^ | 2·9 | 0 | **<0·0001** |
| Diabetes (%) | 27·7^‡^ | 18·9^‡^ | 26·1^‡^ | 4·0 | **0·0064** |
| Hypertension (%) | 92·6^†‡^ | 73·0^‡^ | 75·4^‡^ | 14·0 | **<0·0001** |
| SAHS (%) | 13·8^‡^ | 8·1 | 24·6^‡^ | 0 | **0·0010** |
| Dyslipidemia (%) | 43·6 | 35·1 | 50·7^‡^ | 26·0 | **0·043** |
| Uric acid(μmol/l, n=194) | 460·6±137·7*^†‡^ | 401·5±109·7 | 382·6±83·6 | 326·9±70·6 | **<0·0001** |
| HsCRP(mg/l, n=189) | 1·38(0·66,3·89)^‡^ | 1·11(0·48,2·94) | 1·45(0·79,2·83)^‡^ | 0·66(0·38,1·14) | **0·011** |
| Hematocrit (%, n=190) | 0·43±0·05 | 0·41±0·04 | 0·43±0·06 | 0·42±0·04 | 0·25 |
| Hemoglobin(g/l, n=190) | 148±18 | 139±15^†^ | 150±17 | 143±14 | **0·016** |
| Creatinine(umol/l, n=198) | 95·2±22·4^†‡^ | 90·0±19·8 | 82·0±14·3 | 78·5±13·8 | **<0·0001** |
| GFR(ml/min/1·73m^2^, n=198) | 79±19^†‡^ | 77±23^†‡^ | 91±14 | 90±14 | **<0·0001** |
| Blood glucose(mmol/l, n=197) | 6·1±2·1 | 6·3±2·7 | 6·2±2·7 | 5·1±0·6 | 0·13 |
| NT-proBNP (pg/ml, n=170) | 314(148,847)^†‡^ | 707(218,1964)^†‡^ | 24(16,37) | 37(17,84) | **<0·0001** |
| ACEI/ARB (%) | 74·5^†‡^ | 59·5^‡^ | 39·1^‡^ | 2·0 | **<0·0001** |
| β-blocker (%) | 70·2^†‡^ | 64·9^‡^ | 44·9^‡^ | 10·0 | **<0·0001** |
| Ca^2+^ antagonists (%) | 43·6^‡^ | 21·6 | 31·9^‡^ | 6·0 | **<0·0001** |
| Statin (%) | 31·9^‡^ | 24·3^‡^ | 24·6^‡^ | 4·0 | **0·0023** |
| Aspirin (%) | 17·0 | 16·2 | 17·4 | 6·0 | 0·28 |
| Diuretic (%) | 71·3^†‡^ | 51·4^†‡^ | 8·7 | 0 | **<0·0001** |
| **CMR data** |  |  |  |  |  |
| LVEF (%) | 56±6^†‡^ | 57±5^†‡^ | 60±5 | 62±4 | **<0·0001** |
| Heart rate (bpm) | 70±12 | 69±14 | 71±12 | 69±11 | 0·86 |
| Cardiac index (ml/m^2^) | 3·5±0·8 | 3·2±0·8 | 3·2±0·8 | 3·3±0·8 | **0·038** |
| LAV (ml) | 90±34^†‡^ | 87±36^†‡^ | 72±16^‡^ | 58±14 | **<0·0001** |
| LAVi (ml/m^2^) | 44·2±17·2*^†‡^ | 54·1±23·5^†‡^ | 35·1±7·2 | 35·6±9·4 | **<0·0001** |
| LVEDD (mm) | 56±6*^†‡^ | 50±6 | 51±6^‡^ | 47±4 | **<0·0001** |
| LVEDDi (mm/m^2^) | 27·7±3·2*^†^ | 30·6±4·1^†‡^ | 24·8±3·1^‡^ | 28·6±2·3 | **<0·0001** |
| LVEDVi (ml/m^2^) | 91·8±21·1*^†‡^ | 81·0±19·5 | 74·9±13·5 | 74·9±11·4 | **<0·0001** |
| LVESVi (ml/m^2^) | 41·2±13·0*^†‡^ | 35·1±11·6 | 30·1±7·6 | 28·7±6·2 | **<0·0001** |
| LVMi(g/m^2.7^) | 34·4±10·5*^†‡^ | 22·0±6·0 | 25·8±5·4^‡^ | 18·1±3·0 | **<0·0001** |
| GLS (%) | -11·9±2·9*^†‡^ | -13·8±2·5^†‡^ | -16·0±2·8 | -16·3±2·1 | **<0·0001** |
| GCS (%) | -13·4±3·3*^†‡^ | -14·9±3·0^†‡^ | -18·2±2·7 | -18·9±1·7 | **<0·0001** |
| GRS (%) | 20·7±7·3^†‡^ | 24·2±7·4^†‡^ | 31·6±7·1 | 33·3±4·8 | **<0·0001** |
| sGLSR (/s) | -0·66±0·14^†‡^ | -0·72±0·18^†‡^ | -0·82±0·13 | -0·83±0·15 | **<0·0001** |
| sGCSR (/s) | -0·78±0·17^†‡^ | -0·80±0·20^†‡^ | -0·95±0·15 | -0·97±0·16 | **<0·0001** |
| sGRSR (/s) | 1·19±0·43^†‡^ | 1·29±0·44^†‡^ | 1·61±0·35 | 1·70±0·37 | **<0·0001** |
| eGLSR (/s) | 0·50±0·17*^†‡^ | 0·61±0·25^‡^ | 0·69±0·17^‡^ | 0·79±0·21 | **<0·0001** |
| eGCSR (/s) | 0·54±0·19^†‡^ | 0·61±0·29^†‡^ | 0·76±0·19^‡^ | 0·87±0·20 | **<0·0001** |
| eGRSR (/s) | -0·95±0·43^†‡^ | -1·09±0·53^†‡^ | -1·62±0·60 | -1·72±0·42 | **<0·0001** |
| Presence of LGE (n=115) | 30·0 (21/70) | 10·0 (2/20) | 13·6 (3/22) | 0 (0/3) | 0·15 |
| LGE percentages (%/LV) | 0·6±1·1 | 0·2±0·9 | 0·3±0·9 | 0 | 0·49 |
| Native T1 (ms, n=83) | 1329±97 | 1315±183 | 1275±72 | - | 0·24 |
| ECV (%, n=83) | 30·8±5·0^†^ | 29·8±3·3 | 26·2±3·9 | - | **0·0023** |

Table S4 Baseline and CMR data in patients without coronary artery disease

Values are given as mean ± SD or percentages. The P-values indicate the statistic power among all four group. Bold P-values indicate a significance level of <0·05. * p<0·05 vs. patients with HFpEF and normal weight; †, p<0·05 vs. patients with obesity; ‡, p<0·05 vs. controls using post hoc Bonferroni analysis. HFpEF, heart failure with preserved ejection fraction; BP, blood pressure; PV, plasma volume; SAHS, obstructive sleep apnea hypopnea syndrome; Hs-CRP, hypersensitive-C reactive protein; GFR, glomerular filtration rate; NT-proBNP, N-terminal pro–brain natriuretic peptide; ACEI/ARB, Angiotensin-Converting Enzyme Inhibitors/Angiotensin Receptor Blockers. CMR, cardiovascular magnetic resonance; LV, left ventricular; LA, left atrial; EF, ejection fraction; LVEDD, LV end-diastolic diameter, LVEDDi, LVEDD index; EDVi/ESVi, end-diastole/systole volume index; LVMi, LV end-diastolic mass/height^2.7^; LAV, LA maximal volume; LAVi, LAV index; GLS, GCS, and GRS, global longitudinal, circumferential, radial strain; sGLSR, sGCSR and sGRSR, systolic GLS, GCS, GRS rate; eGLSR, eGCSR and eGRSR, early-diastolic GLS, GCS, GRS rate; LGE, late gadolinium enhancement; ECV, extracellular volume fraction.

Table S5 Comparisons of CMR data in HFpEF patients without coronary artery disease using analysis of covariance

| **Variables** | **Patients with HFpEF and obesity**  **（n=94）** | **Patients with HFpEF and normal weight**  **(n=37)** | **P-value** |
| --- | --- | --- | --- |
| LVEF (%) | 56±1 | 56±1 | 0·84 |
| Heart rate (bpm) | 69±1 | 70±2 | 0·85 |
| Cardiac index (ml/m^2^) | 3·4±0·1 | 3·5±0·1 | 0·53 |
| LAV (ml) | 96±3 | 72±6 | **0·0007** |
| LAVi (ml/m^2^) | 47·9±1·8 | 44·5±3·1 | 0·36 |
| LVEDD (mm) | 56±1 | 52±1 | **0·010** |
| LVEDDi (mm/m^2^) | 27·6±0·4 | 30·8±0·6 | **0·0001** |
| LVEDVi (ml/m^2^) | 88·5±2·1 | 89·5±3·6 | 0·83 |
| LVESVi(kg/m^2^) | 39·4±1·3 | 39·6±2·3 | 0·95 |
| LVMi(g/m^2.7^) | 32·5±0·9 | 27·0±1·5 | **0·0039** |
| GLS (%) | -12·0±0·3 | -13·5±0·5 | **0·021** |
| GCS (%) | -13·6±0·3 | -14·6±0·6 | 0·13 |
| GRS (%) | 21·1±0·8 | 23·2±1·3 | 0·20 |
| sGLSR (/s) | -0·67±0·02 | -0·71±0·03 | 0·18 |
| sGCSR (/s) | -0·77±0·02 | -0·83±0·03 | 0·12 |
| sGRSR (/s) | 1·14±0·04 | 1·28±0·07 | 0·10 |
| eGLSR (/s) | 0·50±0·02 | 0·60±0·04 | **0·030** |
| eGCSR (/s) | 0·54±0·03 | 0·60±0·04 | 0·28 |
| eGRSR (/s) | -0·95±0·05 | -1·10±0·09 | 0·17 |
| ECV (%, n=83) | 31·0±0·7 | 29·4±1·3 | 0·32 |

Adjustment of confounding baseline factors, including age, atrial fibrillation in patients with HFpEF and obesity and patients with HFpEF and normal weight using analysis of covariance. Values are given as mean ± SE. Bold P-values indicate a significance level of <0.05. HFpEF, heart failure with preserved ejection fraction; CMR, cardiovascular magnetic resonance; LV, left ventricular; LA, left atrial; EF, ejection fraction; LVEDD, LV end-diastolic diameter, LVEDDi, LVEDD index; EDVi/ESVi, end-diastole/systole volume index; LVMi, LV end-diastolic mass/height^2.7^; LAV, LA maximal volume; LAVi, LAV index; GLS, GCS, and GRS, global longitudinal, circumferential, radial strain; sGLSR, sGCSR and sGRSR, systolic GLS, GCS, GRS rate; eGLSR, eGCSR and eGRSR, early-diastolic GLS, GCS, GRS rate; LGE, late gadolinium enhancement; ECV, extracellular volume fraction.

Table S6 Correlation analysis of LV strain parameters with presence of LGE and ECV in patients with HFpEF and obesity

|  | **Presence of LGE (n=82)** | |  | **ECV (%，n=58)** | |
| --- | --- | --- | --- | --- | --- |
|  | ρ | P-value |  | r | P-value |
| GLS (%) | -0·208 | 0·061 |  | 0·125 | 0·35 |
| GCS (%) | -0·238 | **0·032** |  | -0·008 | 0·95 |
| GRS (%) | -0·205 | 0·065 |  | 0·010 | 0·94 |
| sGLSR (/s) | -0·136 | 0·22 |  | 0·072 | 0·59 |
| sGCSR (/s) | -0·163 | 0·14 |  | -0·218 | 0·10 |
| sGRSR (/s) | -0·187 | 0·093 |  | -0·158 | 0·24 |
| eGLSR (/s) | -0·263 | **0·017** |  | 0·104 | 0·44 |
| eGCSR (/s) | -0·346 | **0·0014** |  | -0·036 | 0·79 |
| eGRSR (/s) | -0·269 | **0·015** |  | -0·028 | 0·84 |
| E/E’ | 0·217 (n=32) | 0·23 |  | 0·267 (n=26) | 0·19 |

Strain parameters were expressed as absolute values, and the lower values meant worse cardiac function. GLS, GCS, and GRS, global longitudinal, circumferential, radial strain; sGLSR, sGCSR and sGRSR, systolic GLS, GCS, GRS rate; eGLSR, eGCSR and eGRSR, early-diastolic GLS, GCS, GRS rate; LGE, late gadolinium enhancement; ECV, extracellular volume fraction; E/E’, early-diastolic mitral inflow velocity/mean mitral annular peak early diastolic velocity.

Table S7 Correlation analysis of LV strain parameters with presence of LGE and ECV in all patients

|  | **Presence of LGE (n=140)** | |  | **ECV (%，n=98)** | |
| --- | --- | --- | --- | --- | --- |
|  | ρ | P-value |  | r | P-value |
| GLS (%) | -0·300 | **0·0003** |  | -0·122 | 0·23 |
| GCS (%) | -0·264 | **0·0016** |  | -0·217 | **0·032** |
| GRS (%) | -0·250 | **0·0029** |  | -0·219 | **0·030** |
| sGLSR (/s) | -0·256 | **0·0022** |  | -0·134 | 0·19 |
| sGCSR (/s) | -0·166 | 0·050 |  | -0·234 | **0·020** |
| sGRSR (/s) | -0·207 | **0·014** |  | -0·254 | **0·011** |
| eGLSR (/s) | -0·290 | **0·0005** |  | -0·048 | 0·64 |
| eGCSR (/s) | -0·355 | **<0·0001** |  | -0·075 | 0·47 |
| eGRSR (/s) | -0·300 | **0·0003** |  | -0·183 | 0·071 |
| E/E’ | 0·146 (n=47) | 0·33 |  | 0·284 (n=39) | 0·080 |

Strain parameters were expressed as absolute values, and the lower values meant worse cardiac function. GLS, GCS, and GRS, global longitudinal, circumferential, radial strain; sGLSR, sGCSR and sGRSR, systolic GLS, GCS, GRS rate; eGLSR, eGCSR and eGRSR, early-diastolic GLS, GCS, GRS rate; LGE, late gadolinium enhancement; ECV, extracellular volume fraction; E/E’, early-diastolic mitral inflow velocity/mean mitral annular peak early diastolic velocity.

Table S8 Intra and Inter-observer reproducibility for CMR-FT derived strain parameters

| **Variables** | **Intra-observer** | | |  | **Inter-observer** | | |
| --- | --- | --- | --- | --- | --- | --- | --- |
|  | ICC | 95% CI | CoV (%) |  | ICC | 95% CI | CoV (%) |
| GLS (%) | 0·998 | 0·995-0·999 | 1·7 |  | 0·939 | 0·705-0·981 | 7·6 |
| GCS (%) | 0·992 | 0·979-0·997 | 2·9 |  | 0·966 | 0·917-0·986 | 5·9 |
| GRS (%) | 0·994 | 0·985-0·998 | 3·8 |  | 0·974 | 0·936-0·990 | 8·0 |
| sGLSR (/s) | 0·987 | 0·969-0·995 | 4·6 |  | 0·930 | 0·832-0·972 | 10·7 |
| sGCSR (/s) | 0·996 | 0·990-0·998 | 2·6 |  | 0·948 | 0·874-0·979 | 9·3 |
| sGRSR (/s) | 0·982 | 0·955-0·993 | 6·7 |  | 0·964 | 0·913-0·986 | 9·3 |
| eGLSR (/s) | 0·989 | 0·972-0·996 | 5·5 |  | 0·970 | 0·925-0·988 | 9·2 |
| eGCSR (/s) | 0·998 | 0·996-0·999 | 2·4 |  | 0·956 | 0·895-0·982 | 11·8 |
| eGRSR (/s) | 0·997 | 0·993-0·999 | 3·6 |  | 0·950 | 0·877-0·980 | 14·5 |

CMR-FT, cardiovascular magnetic resonance-feature tracking; ICC, intraclass correlation coefficient; CI, confidence interval; GLS, GCS, and GRS, global longitudinal, circumferential, radial strain; sGLSR, sGCSR and sGRSR, systolic GLS, GCS, GRS rate; eGLSR, eGCSR and eGRSR, early-diastolic GLS, GCS, GRS rate.

Figure S1 Comparison of subclinical cardiac function in four groups.


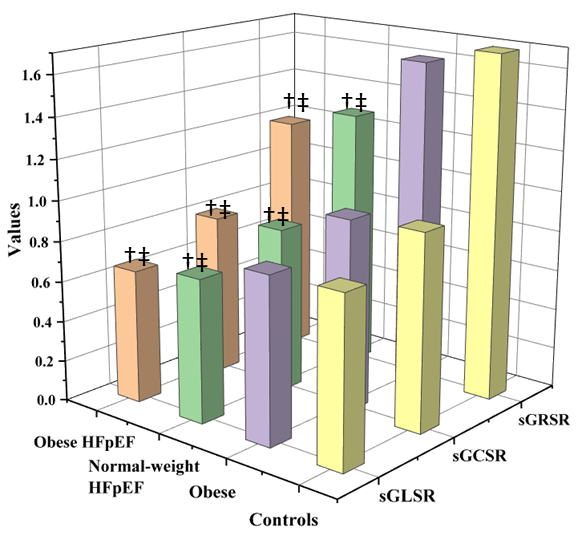


HFpEF, heart failure with preserved ejection fraction; sGLSR/sGCSR/sGRSR, systolic global longitudinal, circumferential, radial strain rate. Data are presented as bars with mean. Strain parameters were expressed as absolute values, and the lower values meant worse cardiac function. †, p<0·05 vs. patients with obesity; ‡, p<0·05 vs. controls.

Figure S2 Regression lines and Bland Altman plots.


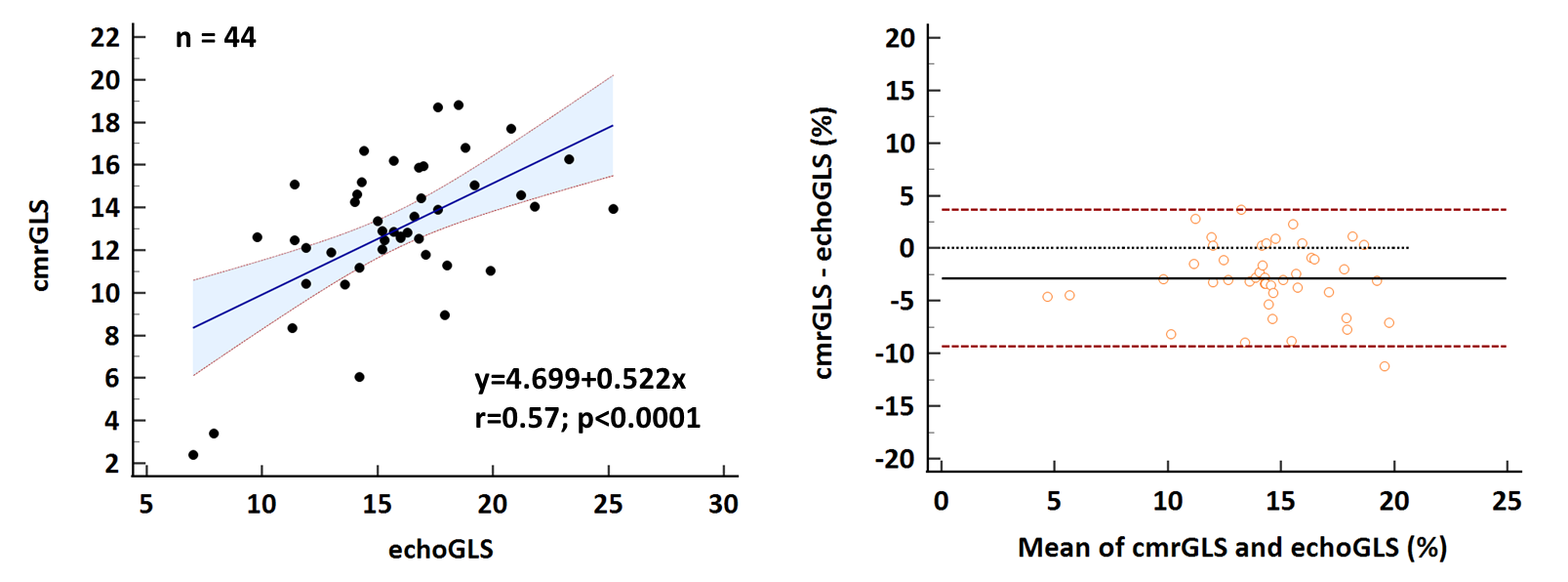


GLS by CMR feature tracking was significantly correlated with that by echocardiography speckle tracking, and the blue shaded regions represented the 95% prediction intervals. Bland Altman plot showed limits of agreement between different software for GLS as well. Strain parameters were expressed as absolute values, and the lower values meant worse cardiac function. echoGLS, echocardiography global longitudinal strain; cmrGLS, cardiovascular magnetic resonance GLS.

Figure S3 Bland–Altman plots for intra- and interobserver variability of CMR strain parameters .


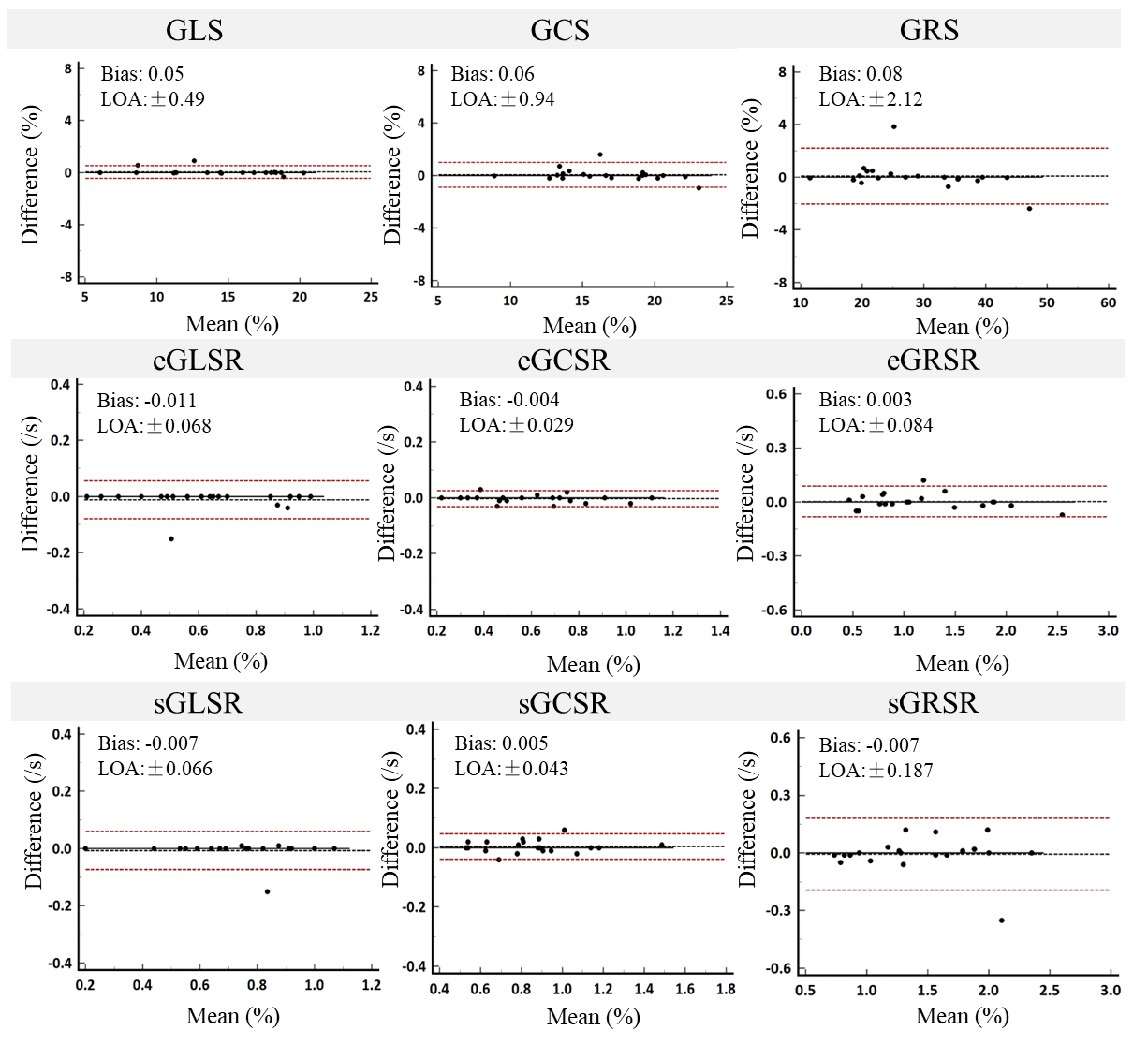


A. intra-observer variability of CMR-FT derived strain parameters.


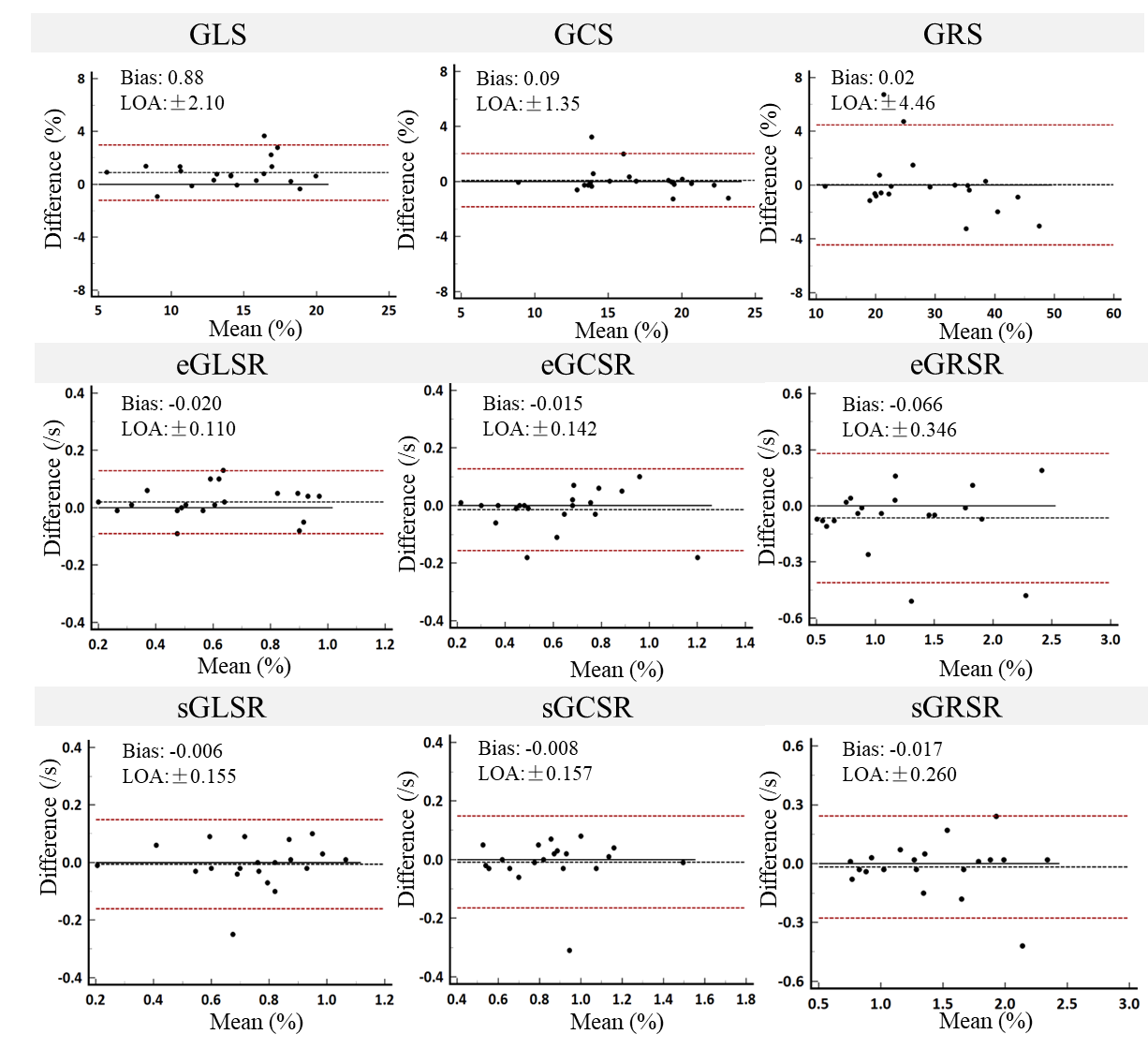


B. inter-observer variability of CMR-FT derived strain parameters.

GLS, GCS, and GRS, global longitudinal, circumferential, radial strain; sGLSR, sGCSR and sGRSR, systolic GLS, GCS, GRS rate; eGLSR, eGCSR and eGRSR, early-diastolic GLS, GCS, GRS rate, CMR-FT, cardiac magnetic resonance- feature tracking; LOA, limits of agreement.
