# Supplementary material for: Clinical features, myocardial strain and tissue characteristics of heart failure with preserved ejection fraction in patients with obesity: A prospective cohort study
Source: eClinicalMedicine. 2022 Nov 3;55:101723. doi: 10.1016/j.eclinm.2022.101723 (PMC9646878; doi:10.1016/j.eclinm.2022.101723)
Supplement: Multimedia component 2 [file mmc2.docx]

**Tables**

Table S1. Echocardiography data

Table S2 Baseline and CMR data in patients without atrial fibrillation

Table S3 Comparisons of CMR data in HFpEF patients without atrial fibrillation using analysis of covariance

Table S4 Baseline and CMR data in patients without coronary artery disease

Table S5 Comparisons of CMR data in HFpEF patients without coronary artery disease using analysis of covariance

Table S6 Correlation analysis of LV strain parameters with presence of LGE and ECV in patients with HFpEF and obesity

Table S7 Correlation analysis of LV strain parameters with presence of LGE and ECV in all patients

Table S8 Intra and Inter-observer reproducibility for CMR-FT derived strain parameters

**Figures**

Figure S1 Comparison of subclinical cardiac function in four groups.

Figure S2 Regression lines and Bland Altman plots.

Figure S3 Bland–Altman plots for intra- and interobserver variability of CMR strain parameters.
